# Werner syndrome RECQ helicase participates in and directs maintenance of the protein complexes of constitutive heterochromatin in proliferating human cells

**DOI:** 10.18632/aging.206132

**Published:** 2024-10-17

**Authors:** Pavlo Lazarchuk, Matthew Manh Nguyen, Crina M. Curca, Maria N. Pavlova, Junko Oshima, Julia M. Sidorova

**Affiliations:** 1 Department of Laboratory Medicine and Pathology, University of Washington, Seattle, WA 98195, USA; 2 Present address: Fred Hutchinson Cancer Center, Seattle, WA 98109, USA; 3 Present address: Parse Biosciences, Seattle, WA 98109, USA

**Keywords:** Werner progeria, heterochromatin, senescence, nuclear lamina, satellite repeats

## Abstract

Werner syndrome of premature aging is caused by mutations in the WRN RECQ helicase/exonuclease, which functions in DNA replication, repair, transcription, and telomere maintenance. How the loss of WRN accelerates aging is not understood in full. Here we show that WRN is necessary for optimal constitutive heterochromatin levels in proliferating human fibroblasts. Locally, WRN deficiency derepresses SATII pericentromeric satellite repeats but does not reduce replication fork progression on SATII repeats. Globally, WRN loss reduces a subset of protein-protein interactions responsible for the organization of constitutive heterochromatin in the nucleus, namely, the interactions involving Lamin B1 and Lamin B receptor, LBR. Both the mRNA level and subcellular distribution of LBR are affected by WRN deficiency, and unlike the former, the latter phenotype does not require WRN catalytic activities. The phenotypes of heterochromatin disruption seen in WRN-deficient proliferating fibroblasts are also observed in WRN-proficient fibroblasts undergoing replicative or oncogene-induced senescence. WRN interacts with histone deacetylase 2, HDAC2; WRN/HDAC2 association is mediated by heterochromatin protein alpha, HP1α, and WRN complexes with HP1α and HDAC2 are downregulated in senescing cells. The data suggest that the effect of WRN loss on heterochromatin is separable from senescence program, but mimics at least some of the heterochromatin changes associated with it.

## INTRODUCTION

Werner syndrome of premature aging, WS, is caused by loss of function mutations in the RECQ helicase/exonuclease WRN [1]. WRN is one of the five RECQ helicases in human cells and, as other RECQs, is involved in virtually every aspect of DNA metabolism [2]. WRN facilitates replication fork progression in S phase [3, 4], and in this role, it may be comparatively more important for the difficult-to-replicate regions of the genome such as fragile sites and inverted repeats [5–7], likely owing to its ability to unwind unusual DNA structures such as G-quadruplexes [8–10], and unwind and degrade RNA:DNA hybrids [11–14]. How these and other activities of WRN come together to accelerate cellular aging and senescence is not understood in full.

Derepression of constitutive heterochromatin, CH, is one of the hallmark phenotypes of aging and senesced cells [15, 16], and it has been argued that WRN deficiency is one of the examples that demonstrate that loss of CH silencing causes cellular aging, because WRN loss expedites both CH loss and senescence in cultured mesenchymal stem cells, MSCs [17]. Nevertheless, it remains to be determined why WRN loss affects CH. Zhang et al. [17] found WRN co-immunoprecipitating with the histone lysine methyltransferase SUV39H1 and heterochromatin protein HP1α (CBX5) which, respectively, generate and bind to the repressive mark H3K9me3; and Lachapelle et al. [18] observed that WRN co-immunoprecipitated with the nuclear lamina component Lamin B1 in a DNA-dependent manner. However, the function(s) of these interactions is still unclear. If WRN does act on CH directly, it remains to be determined which of its activities WRN lends to the assembly and/or functioning of heterochromatin. In the absence of this knowledge, it is still possible that WRN deficiency in MSCs precipitates loss of heterochromatin indirectly, e.g., by promoting senescence through accelerated telomere attrition.

The deacetylases HDAC1 and HDAC2 are involved in the establishment of repressive chromatin genome-wide, including CH and its subset, pericentromeric CH (PCH) [19–21]. HDAC2 was found in complex with SUV39H1 [22] and Lamin A/C [23], and an early study detected HDAC2 but not HDAC1 in a multiprotein complex specific to senescent cells [24]. We originally identified a functional interaction between WRN and HDAC1 at stalled replication forks and found that WRN, HDAC1, and HDAC2, though detectable on replicating DNA, did not move with the replication fork but remained associated with maturing chromatin for hours after replication [25]. A fraction of WRN co-immunoprecipitated with HDAC2 and HDAC1, but WRN presence in either of the HDAC1 or HDAC2 immunoprecipitates depended on HDAC2 presence in the lysates.

Here, we demonstrate that WRN and HDAC2 associate with HP1α and depend on it for their mutual proximity in the cell. WRN, HDAC2, and HP1α associations are suppressed during replicative or oncogene-induced senescence. WRN is involved in CH maintenance in proliferating, telomerase-positive fibroblasts by positively regulating the assemblies that sequester heterochromatin at the nuclear periphery for silencing and that involve associations of heterochromatin complexes with Lamin B1 and Lamin B receptor, LBR. In particular, WRN supports proper levels of LBR by promoting its transcription and enrichment within the nuclei.

## RESULTS

### WRN associates with HDAC2

**Figure 1:**
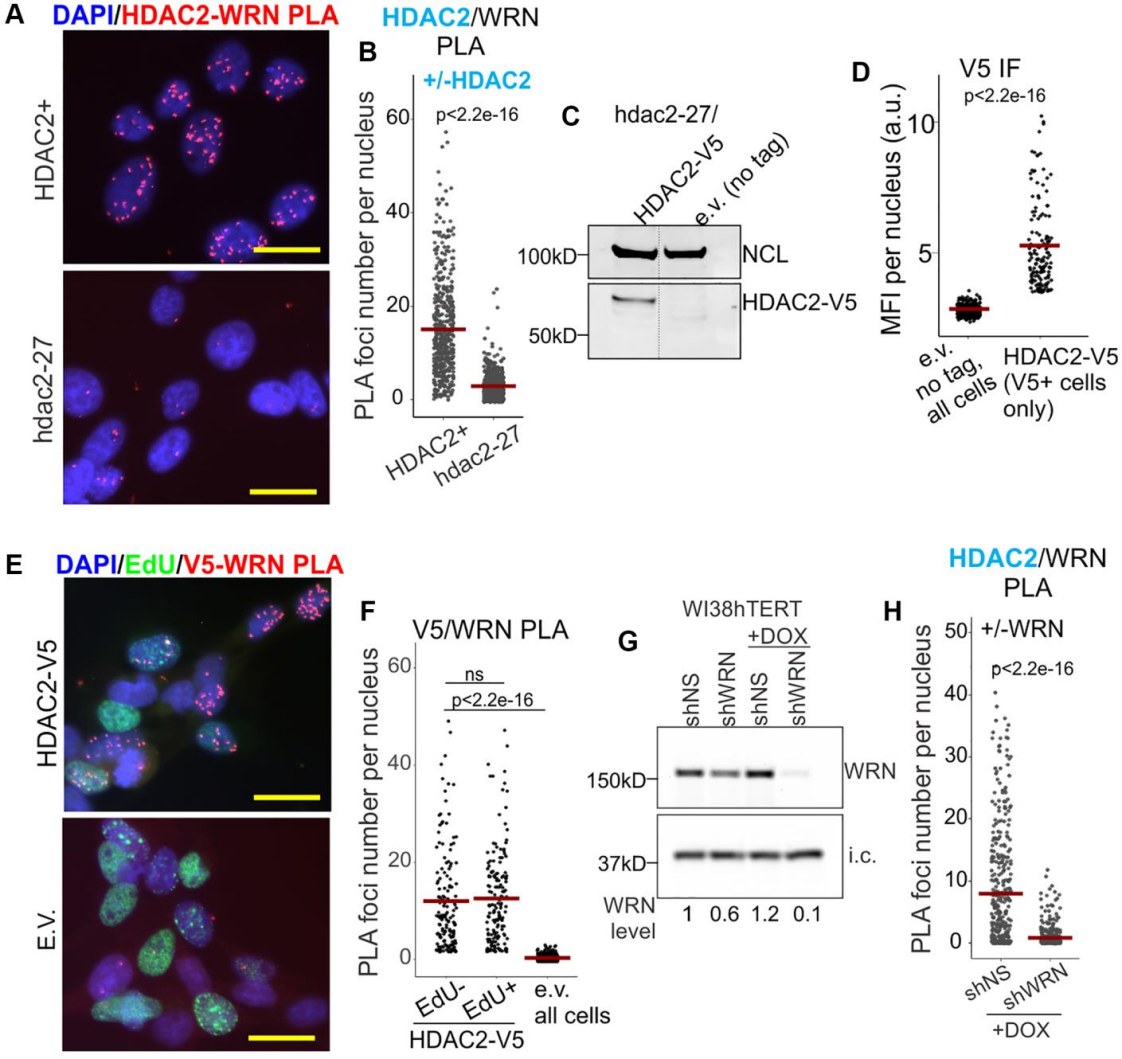
**WRN associates with HDAC2.** (**A**) Representative image of HDAC2/WRN PLA in the isogenic HDAC2+ and hdac2-27 null derivatives of GM639. Scale, 20 µm. (**B**) Quantitation of HDAC2/WRN PLA performed as in (**A**). (**C**) Hdac2-27 cells were stably transfected with HDAC-V5 transgene or empty vector (tag-less) and analyzed by Western blotting with antibodies to NCL (internal control) and V5. (**D**) Quantitation of IF *in situ* performed with V5 antibody on the same cells as in (**C**). MFI, mean fluorescence intensity, a.u., arbitrary units. (**E**) Representative image of V5/WRN PLA performed on the same cells as in (**C**, **D**) that were labeled for 30 min with EdU prior to harvest. EdU incorporation was visualized by Clicking to Alexa 488 azide. Scale, 20 µm. (**F**) Quantitation of V5/WRN PLA performed as in (**E**). V5/WRN signals of EdU-positive and EdU-negative cells are plotted separately. (**G**) WI38hTERT cells were stably transfected with the indicated shRNA-expressing constructs and treated with 100 ng/ml doxycycline for 5 days prior to Western blotting with WRN antibody. i.c., internal control (the STING protein). I.c.-normalized WRN levels are shown relative to shNS w/o DOX. (**H**) Quantitation of HDAC2/WRN PLA performed with the same cells as in (**F**). Crossbars in jitter plots are distribution means. Here and elsewhere, see Methods for details of *p*-value calculations.

We developed PLA assays to visualize WRN/HDAC2 and WRN/HDAC1 physical proximities *in situ*. Robust WRN/HDAC2 PLA signal was detected in the GM639 cell line of SV40-immortalized fibroblasts and was dramatically reduced in the isogenic hdac2-27 null derivative (Figure 1A, 1B, also see Supplementary Figure 1A for a zoomed-out, split channel view of the same cells). A V5-tagged version of HDAC2 was introduced into hdac2-27 cells (Figure 1C, 1D), and a V5/WRN PLA signal was observed in these cells both in S phase and outside it, as identified by pulse-labeling with EdU (Figure 1E, 1F and Supplementary Figure 1B for a zoomed-out view by channel). Thus, the WRN/HDAC2 association was not limited to replicating chromatin. WRN-dependence of the WRN/HDAC2 PLA signal was also confirmed with a derivative of WI38hTERT fibroblasts in which WRN was stably depleted by doxycycline-inducible shRNA expressed from a lentiviral vector (Figure 1G, 1H; unless stated otherwise, here and elsewhere WI38hTERT cells were maintained in a WRN-depleted state for multiple passages to approximate a steady state of WRN deficiency).

To test for involvement of HDAC1, we used clonally derived control and hdac1 null normal human dermal fibroblasts (NHDFs) in which HDAC1 was disrupted (Supplementary Figure 1C) using CRISPR/Cas9 as described before [26]. We detected a more robust signal of PLA foci of WRN/HDAC2 compared to WRN/HDAC1, the latter measuring virtually at a background level (Supplementary Figure 1D, 1E). The WRN/HDAC2 signal was once again present in cells outside of S phase and was not reduced in hdac1 null cells (Supplementary Figure 1E), suggesting that the WRN/HDAC2 association does not require HDAC1. A mild elevation of the HDAC2/WRN signal in hdac1 null compared to the HDAC1 wt control is consistent with the compensatory increase in HDAC2 levels upon HDAC1 disruption, as we and others observed before [26, 27]. Together, these observations led us to focus on the WRN/HDAC2 association for further study.

### WRN and HDAC2 associate with heterochromatin protein 1 alpha (HP1α) independently of each other and depend on it for mutual proximity

Given that WRN/HDAC2 association was not limited to S phase cells, we reasoned that the putative function of this association is not centered on replication forks or newly-replicated DNA. Previous studies suggest an alternative substrate for potential physical and functional interaction of WRN and HDAC2: CH, particularly pericentromeric heterochromatin, PCH [17, 19, 22]. WRN and HDAC2 were found to co-immunoprecipitate with heterochromatin protein 1 alpha, HP1α, a major component of CH and PCH [28, 29], albeit the functional significance of these interactions is unknown.

**Figure 2:**
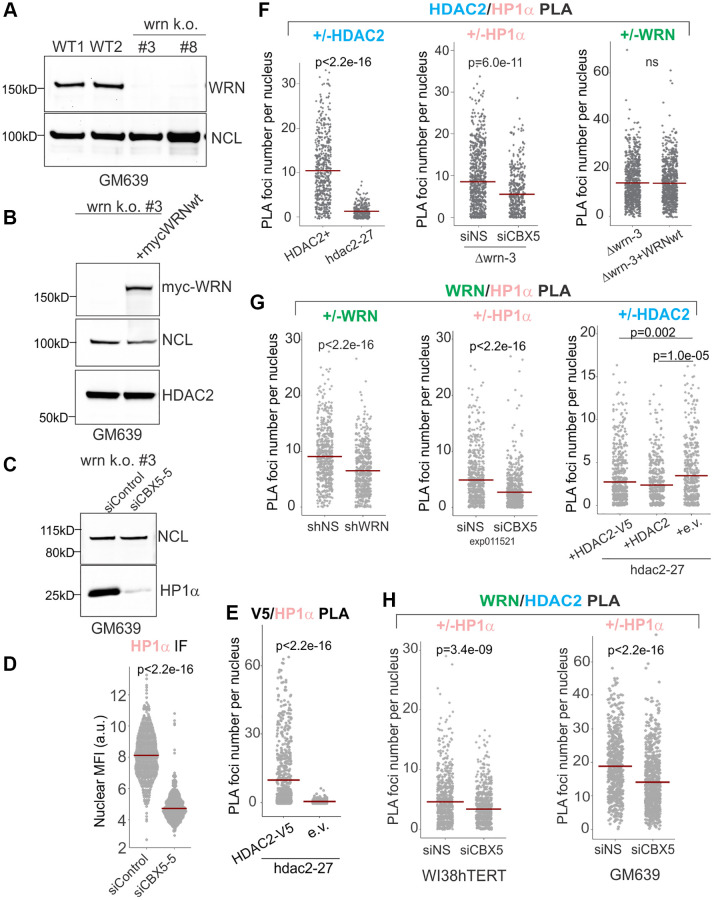
**WRN and HDAC2 proximity is facilitated by HP1α.** (**A**) Clonal isolates of CRISPR/Cas9 knockouts of WRN were generated in the GM639 background. WT1 and WT2 are isogenic WRN wild type controls. WT1 (GM639-Cas9-EV [26]), parent cell line is a GM639 derivative with Cas9 and an empty vector instead of a dgRNA expressing construct. WT2 is one of WRN wild type isolates derived from subcloning of WRN dgRNA-transfected GM639-Cas9 cells. (**B**) Western blot analysis of the *WRN* k.o. #3 cell line (hereafter called Δwrn-3) stably transfected with pLX209-neo-WRN expressing 5xmyc-WRN. (**C**) Δwrn-3 was transfected with non-targeting (siControl) and the *HP1α* gene-targeting (CBX5-5) siRNAs and analyzed by Western blotting at 36 hrs post-transfection. (**D**) WI38hTERT expressing non-targeting shNS were transfected with non-targeting and *HP1α*-targeting (CBX5-5) siRNAs and analyzed by IF *in situ* with HP1α antibody at 36 hrs post-transfection. (**E**) Quantitation of V5/HDAC2 PLA performed in hdac2-27 cells stably transfected with HDAC2-V5 transgene or with empty vector (e.v., no tag). Note that not all transfected cells express HDAC2-V5, thus cells with no PLA signal are expected in the hdac2-27/HDAC2-V5 population. (**F**) Quantitations of HDAC2/HP1α PLA performed in Δwrn-3 and hdac2-27 (left panel), in Δwrn-3 transfected with non-targeting or CBX5-5 siRNAs (center panel), or in Δwrn-3 with or without expression of the WRN transgene (right panel). The graph represents two independent experiments. (**G**) Quantitations of WRN/HP1α PLA performed in WI38hTERT expressing non-targeting shRNA or shRNA against WRN (left panel), in WI38hTERT expressing non-targeting shRNA and also transfected with siRNA against HP1α or a non-targeting control (center panel, the experiment was done at 36 hrs post-transfection); and in hdac2-27 stably expressing the indicated transgenes or an empty vector (right panel, the graph represents three independent experiments). (**H**) Quantitations of WRN/HDAC2 PLA performed in WI38hTERT with siRNA against HP1α or a non-targeting control (left panel) and in WT1 (GM639-Cas9-EV) transfected with the same siRNAs (right panel). Crossbars in graphs are distribution means.

We used several isogenic pairs of cell lines to confirm WRN/HP1α and HDAC2/HP1α associations: (i) the hdac2-27 null and complemented cells described in the previous section, (ii) a CRISPR/Cas9 WRN KO derivative of GM639 Δwrn-3 (Figure 2A) that was stably transfected with a WRN-expressing construct (Figure 2B), (iii) the shRNA-mediated depletion of WRN described in the previous section (Figure 1), and (iv) siRNA-mediated depletion of HP1α (Figure 2C, 2D). Pairwise associations between HDAC2, HP1α, and WRN in cell nuclei were assessed by PLA *in situ*.

We detected a HDAC2-V5 transgene-dependent V5/HP1α PLA signal (Figure 2E) as well as a HDAC2/HP1α PLA signal that was responsive to HDAC2 and HP1α reduction (Figure 2F, left and center panels) but was unaffected by WRN status (Figure 2F, right panel). Likewise, a WRN and HP1α-dependent PLA signal was readily detectable (Figure 2G, left and center panels), and if anything was modestly increased in the absence of HDAC2 (Figure 2G, right panel). Remarkably, depletion of HP1α in WI38hTERT or GM639 cell backgrounds also reduced the HDAC2 to WRN PLA signal (Figure 2H). These data suggest that HP1α provides a physical platform for, or regulates the assembly of a higher order complex within which WRN and HDAC2 are brought into proximity. At the same time, WRN and HDAC2 do not require each other to associate with HP1α. In fact, HDAC2 appears to moderately suppress WRN/HP1α proximity rather than promoting it.

### Senescing cells downregulate WRN, HDAC2, HP1α associations

**Figure 3:**
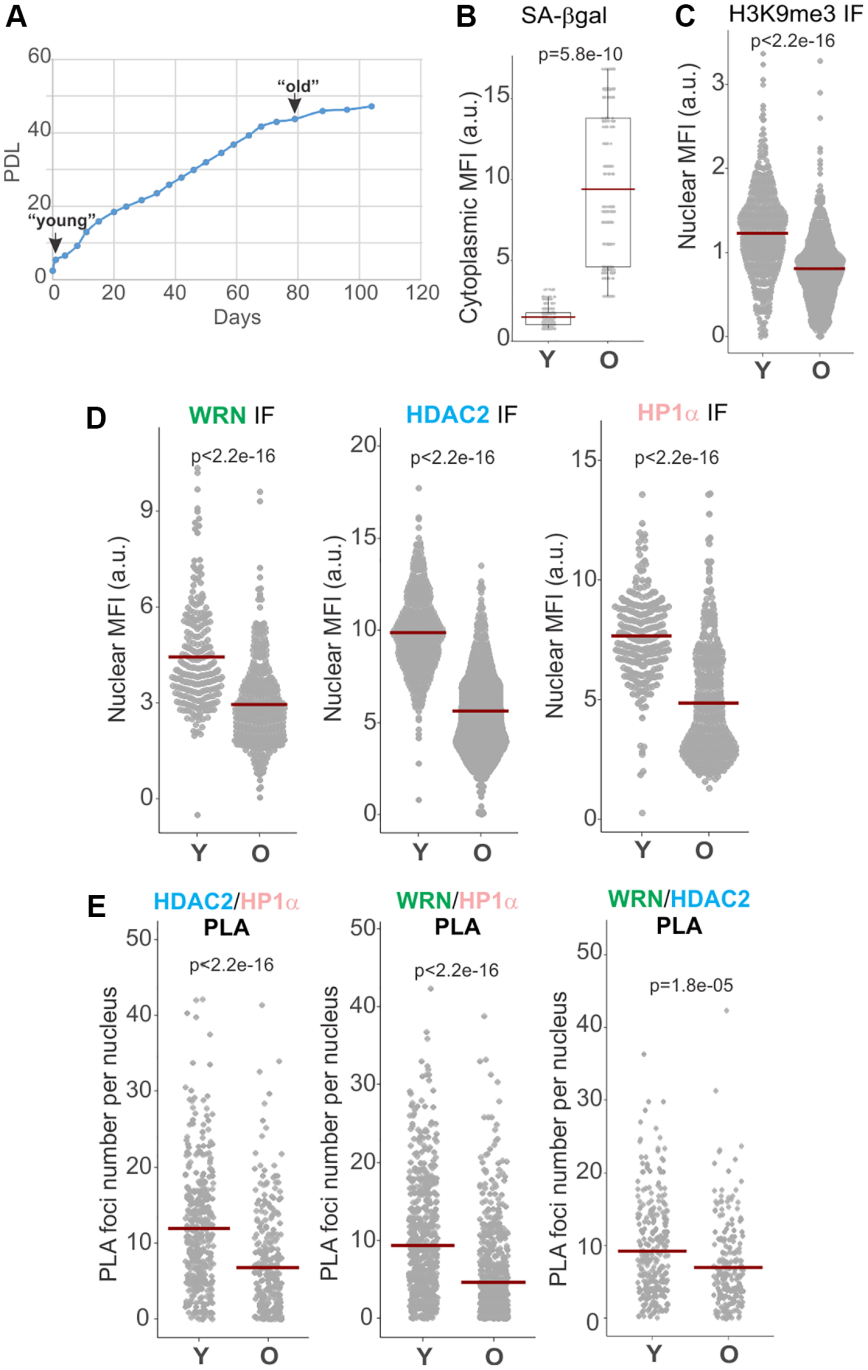
**WRN, HDAC2, and HP1α associations are reduced in replicatively senescing cells.** (**A**) Normal human dermal fibroblasts were passaged to derive early and late passage cultures, which were cryopreserved and subsequently used in the same experiment. (**B**) SA β-gal levels in young (Y) and old (O) fibroblasts were quantified via fluorescent detection and microscopy. Cytoplasmic MFI was measured per digital image, normalized to the number of cells in each image determined by DAPI counterstaining, and plotted. (**C**, **D**) Quantitation of H3K9me3, WRN, HDAC2, and HP1α MFIs in young vs. old fibroblasts determined by IF *in situ*. (**E**) Quantitations of HDAC2/HP1α, WRN/HP1α, and WRN/HDAC2 PLA signals in young vs. old fibroblasts. Graphs in C, D, and E represent two independent experiments each. Crossbars in graphs are distribution means.

Given the findings that HDAC2 [24] and WRN [17] complexes may be altered in senescing cells, we examined how two distinct types of senescence, replicative senescence (RS) and oncogene-induced senescence (OIS) affect the state of WRN, HDAC2, and HP1α associations. Normal human dermal fibroblasts (NHDFs) were serially passaged and cryopreserved throughout to generate early and late population doubling level (PDL) cultures to compare side by side later. The PDL5 (“young”) and PDL47 (“old”) fibroblasts were chosen for comparison (Figure 3A, PDL values correspond to those at the beginning of the experimental series). Old fibroblasts exhibited slower growth but were not yet fully senesced, presenting a heterogeneous population of cells with normal or enlarged nuclei and an overall elevated level of senescence-associated, SA, β-gal staining (Figure 3B) and reduced mean fluorescence intensity (MFI) of H3K9me3 (Figure 3C). Nuclear MFIs of HDAC2, HP1α, and WRN in old cells were reduced compared to proliferating young cells in IF *in situ* assays (Figure 3D). Notably, all three of pairwise associations, HP1α/WRN, HDAC2/WRN, and HDAC2/HP1α, were also reduced in old fibroblasts compared to young (Figure 3E and Supplementary Figure 2). Of note, the total levels of these proteins in whole cell extracts appeared similar between old and confluent young fibroblasts, whereas sub-confluent, proliferating young fibroblasts showed modestly elevated WRN and HP1α levels (Supplementary Figure 3A) when quantified against NCL or GAPDH controls (Supplementary Figure 3B, 3C). The data suggest that WRN, HDAC2, HP1α densities and mutual proximities in the nuclei are downregulated in old primary fibroblasts or undergo changes resulting in lower detectability *in situ*.

**Figure 4:**
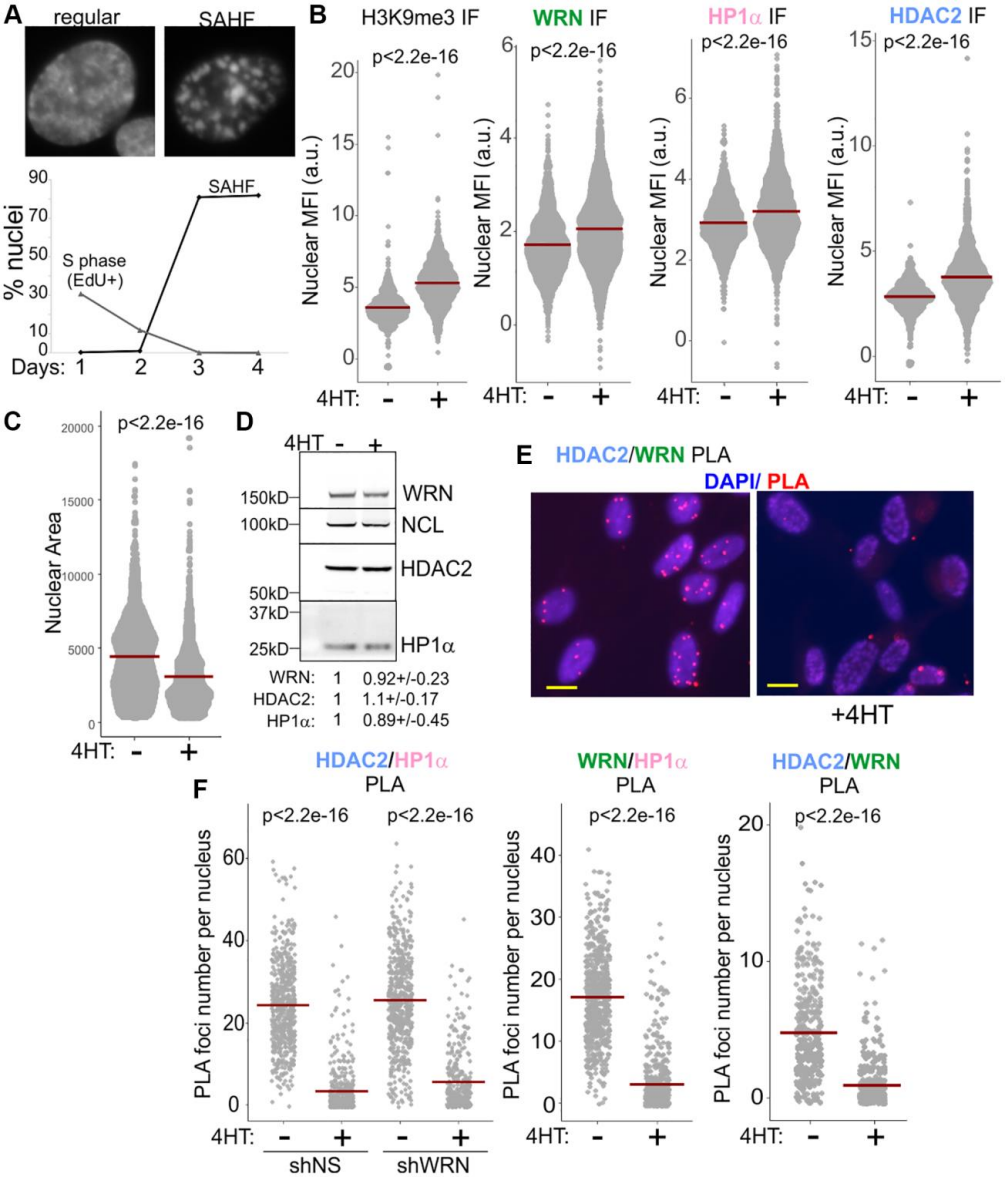
**WRN, HDAC2, and HP1α associations are reduced in RAF oncogene-induced senescence.** (**A**) 4HT-inducible expression of RAF oncogene in WI38hTERT leads to cessation of proliferation and onset of senescence marked by the formation of SAHF after three days. (**B**) Quantitations of H3K9me3 (left panel, three independent experiments), and WRN, HP1α, and HDAC2 (center and right panels, two independent experiments each) nuclear levels in WI38hTERT after 4 days in 4HT compared to the contemporaneous control without 4HT, determined by IF *in situ*. (**C**) Quantitations of nuclear area (in pixels) of WI38hTERT treated with 4HT as in (**B**). The graph represents three independent experiments. (**D**) Total levels of the WRN, HP1α, and HDAC2 proteins in WI38hTERT under the same conditions, determined by Western blotting. Quantitations below the image average three independent experiments. The values were normalized to NCL as loading control and shown relative to no-4HT conditions. (**E**) An example of HDAC2/WRN PLA data in WI38hTERT with and without 4HT. Scale, 10 µm. (**F**) Quantitations of HDAC2/HP1α, WRN/HP1α, and HDAC2/WRN PLA in WI38hTERT after 4 days in 4HT compared to the contemporaneous no-4HT control. The left and center panels represent two independent experiments each, and the right panel represents two biological replicas. Crossbars in graphs are distribution means.

Oncogene-induced senescence, OIS, triggered by the overexpression of oncogenes such as RAF or RAS shares some features with replicative senescence, some of the key differences being a rapid, abrupt onset and the spatial reorganization of chromatin into senescence-associated heterochromatin foci, or SAHF [30], which appears to reflect contraction of the chromosomal territories [31–35] (Figure 4A). This, however, does not indicate a global heterochromatin dismantlement in RAF OIS [36–38], and indeed H3K9me3 is not reduced in OIS cells ([39], also see Figure 4B, left panel). At the same time, CH domains, including pericentromeres, may be selectively relaxed in OIS [40–42]. We used an inducible RAF model system [43] to trigger OIS in WI38hTERT by the addition of 4-HT to the cell media (Figure 4A). Nuclear MFIs of WRN, HP1α, and HDAC2 were higher in OIS cells compared to non-OIS controls (Figure 4B). These values could at least in part be explained by a reduction of nuclear area in OIS (Figure 4C), given the fact that total levels of HDAC2, WRN, and HP1α in whole cell extracts from OIS cells were comparable to those in proliferating cells (Figure 4D). Strikingly, however, the HP1α/WRN, HP1α/HDAC2, and WRN/HDAC2 PLA signals in OIS cells were drastically reduced (Figure 4E, 4F and Supplementary Figure 4), strongly suggesting that mutual associations of these proteins are downregulated in this type of senescence.

### Constitutive heterochromatin maintenance is negatively affected in WRN-deficient proliferating cells

**Figure 5:**
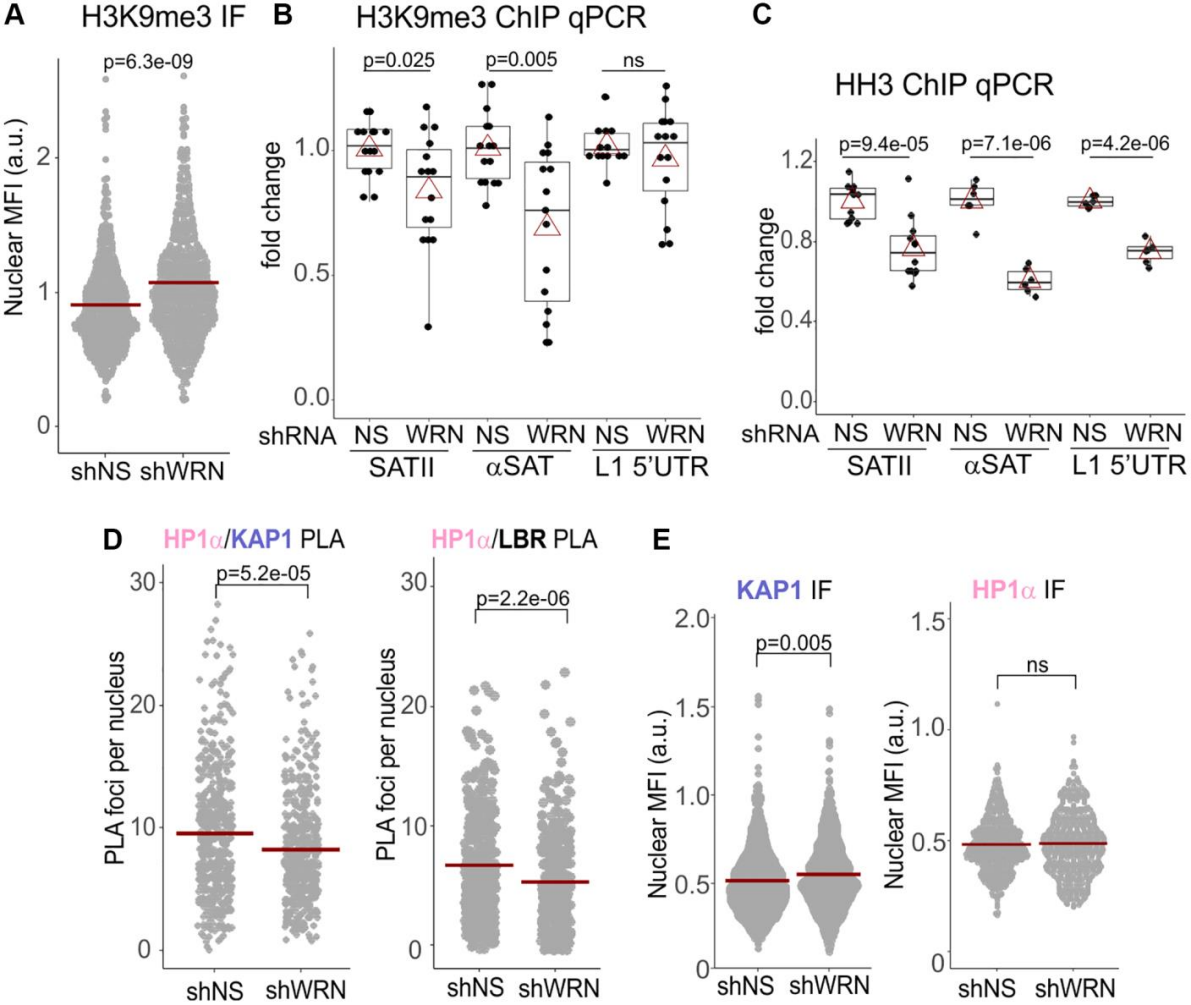
**H3K9me3 heterochromatic mark is reduced on satellite tandem repeats in WRN-depleted cells.** (**A**) Quantitation of IF *in situ* for H3K9me3 levels in WI38hTERT expressing shRNA against WRN or non-specific control (NS). Crossbars are distribution means. The graph represents three independent experiments. (**B**) ChIP qPCR analyses of H3K9me3 levels on the indicated genomic loci, performed in the same cells as in (**A**). The graph summarizes results of five independent experiments with technical triplicates. (**C**) ChIP qPCR analyses of histone H3 levels on the indicated genomic loci, performed in the same cells as in (**A**). The graph summarizes results of three independent experiments with technical triplicates. In (**B**-**C**), red triangles are means, and boxplots mark first and fourth quartiles and the distributions’ medians. (**D**) Quantitations of the indicated PLA *in situ* analyses in WI38hTERT cells expressing shRNA against WRN or control shRNA. Graphs represent two (HP1α/KAP1) and four (HP1α/LBR) independent experiments each. (**E**) Quantitations of the indicated IF *in situ* analyses performed on the same cells as in (**D**). Graphs represent two (KAP1), and three (HP1α) independent experiments each.

We next asked if WRN deficiency is sufficient to elicit CH defects in proliferating cells. The overall H3K9me3 signal intensity *in situ* was somewhat increased in WRN-depleted WI38hTERT compared to the isogenic control (Figure 5A), arguing against a global reduction of this heterochromatin mark. We thus looked at the specific loci commonly monitored as bona fide representatives of CH – the SATII and αSAT classes of satellite repeats that make up pericentromeric CH (PCH), as well as the most abundant class of repeats that constitutes the interspersed CH, LINE1. There was an overall decrease of H3K9me3 on SATII and αSAT, and less so on the LINE1 5′UTR (Figure 5B). These changes were accompanied by reductions of histone H3 occupancies on the same sites (Figure 5C), suggesting a possibility of an increased histone H3 turnover. To follow this up, we looked at SATII expression levels in cells with and without WRN. However, in WI38hTERT, SATII transcripts were difficult to detect (Supplementary Figure 5A, left panel), unless the cells were induced to enter OIS, where, as expected (reviewed in [16]), the level of SATII transcripts was significantly upregulated (Supplementary Figure 5A, right panel). Though WRN-depleted WI38hTERT showed somewhat higher SATII RNA levels both in proliferating and OIS cells, the data did not reach significance (Supplementary Figure 5A). Overall, the data do not indicate a global loss of repressive H3K9me3 mark in WRN deficient proliferating cells.

CH comprises a network of HP1α interactions, including with Lamins and Lamin receptors anchored in the inner nuclear membrane, INM [44]. The latter interactions participate in tethering of heterochromatin to the nuclear periphery [45]. We hypothesized that CH maintenance in WRN-deficient cells is affected through altered interactions of HP1α, and examined its well-known associations with KAP1 and the Lamin B receptor, LBR [28, 46], which are important for heterochromatin assembly (Figure 5D, see Supplementary Figure 6A for PLA controls).

**Figure 6:**
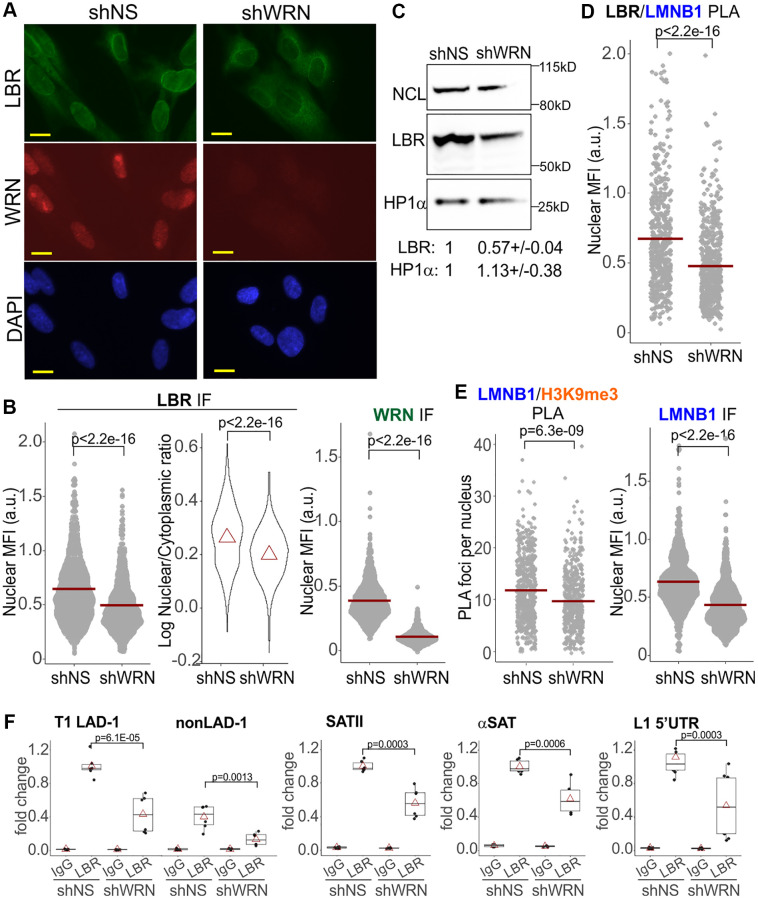
**Reduction of Lamin B1, LBR-associated interactions in WRN-depleted cells.** (**A**) An example of LBR and WRN IF *in situ* in WI38hTERT cells expressing the indicated shRNAs. Scale, 10 µm. (**B**) Quantitations of the indicated IF *in situ* analyses in the same WI38hTERT cells as in (**A**). Left panel, mean nuclear levels of LBR (per cell) background-corrected by the means of the entire fluorescent signal outside the nuclei (per image). Right panel, ratios of mean nuclear to mean cytoplasmic LBR IF signals per cell. Note the log scale. Red triangles are distribution means. The graphs represent four (LBR, left panel), two (LBR, right panel) and over five (WRN) independent experiments each. (**C**) A Western blot of WI38hTERT expressing the indicated shRNAs and probed with antibodies against NCL (internal control), LBR, and HP1α. Quantitations below the image average two independent experiments. The values were normalized to NCL and shown relative to shNS control. (**D**, **E**) Quantitations of the indicated PLA or IF *in situ* analyses in the same WI38hTERT cells as above. The panels represent two (LBR/Lamin B1), three (Lamin B1/H3K9me3), and three (Lamin B1) independent experiments each. Nuclear MFI values were used instead of PLA foci numbers in cases where the latter numbers were too high to robustly count individual foci. (**F**) ChIP qPCR analyses of LBR levels on the indicated genomic loci performed in the same cells as elsewhere in the figure. The graphs summarize results of two independent experiments.

HP1α association with KAP1 was moderately reduced in WRN-depleted WI38hTERT (Figure 5D, left panel). KAP1 nuclear level measured by IF *in situ*, was, if anything, slightly increased by WRN depletion (Figure 5E, left panel). HP1α nuclear level trended as largely unchanged upon WRN depletion (Figure 5E, right panel), though one of the four independent measurements demonstrated a WRN-dependent reduction (Supplementary Figure 6B). It is possible that this variability reflects the complexity of intra- and intermolecular interactions of HP1α, which can affect the accessibility of its epitope *in situ*. No difference in HP1α levels was detected in whole cell extracts of WRN-depleted and control cells by Western blotting (Figure 6C).

HP1α association with LBR trended toward reduction in WRN-deficient cells (Figure 5D, right panel, and Supplementary Figure 6B). LBR, a transmembrane protein, can localize to the inner nuclear membrane, INM, and the endoplasmic reticulum, ER, with the INM typically being its predominant location [47] (Figure 6A). We noted a measurable cytoplasmic staining of LBR, particularly in WRN-deficient WI38hTERT (Figure 6A and Supplementary Figure 6C). Thus, LBR levels within the nuclear perimeter were quantified in two different ways. First, we used the same algorithm as used for other proteins above, which includes normalization to the fluorescence background outside the nuclei and therefore includes the cytoplasmic LBR signal in the denominator (Figure 6B, left panel, nuclear LBR). In addition, the ratio of LBR MFIs within nuclear and cytoplasmic areas was determined using a different algorithm (Figure 6B, right panel). In WRN-deficient cells, both nuclear LBR and the enrichment of LBR in the nucleus vs. the cytoplasm metrics were reduced (Figure 6B). In addition, the LBR protein level was reduced in whole cell extracts of WRN-depleted cells on Western blots (Figure 6C). Consistent with a reduction of the nuclear LBR, Lamin B1/LBR proximity was also significantly reduced without WRN (Figure 6D), accompanied by a reduced nuclear level of Lamin B1 (Figure 6E, right panel), and a reduced Lamin B1/H3K9me3 proximity (Figure 6E, left panel). Lastly, ChIP results showed a reduction in LBR occupancy on SATII and αSAT loci of PCH and LINE1 5′UTR in WRN-deficient cells (Figure 6F). A constitutive lamina-associated domain, LAD, and a non-LAD loci randomly selected from a set identified in [48] demonstrated the expected, respectively higher and lower LBR occupancy, and were also affected by WRN status (Figure 6F). Together, the data suggest that reduction of LBR levels in the nuclei of WRN-deficient cells translates into a functional deficiency in the tethering of CH at the nuclear periphery.

Marked reduction of the nuclear LBR and LMNB1/LBR PLA signals was also seen in WI38hTERT newly depleted of WRN (at day 5 post-induction of shRNA, Supplementary Figure 5B, 5C). Lamin B1 was moderately downregulated and the H3K9me3 mark was upregulated in these cells (Supplementary Figure 5B). Both in chronically and newly WRN-depleted cells, lower LBR levels in the nuclei were not associated with a morphologically distinct subset of cells, as can be seen for example from binning LBR levels by nuclear area (Supplementary Figure 7A). Rather, these values were lower for virtually every nuclear area bin including those containing the bulk of cells in both WRN-depleted and control populations. In addition, nuclear levels of LBR were lower both in S phase and non-S phase WRN-depleted cells (Supplementary Figure 7B). These data argue against a notion that the lower population mean of LBR levels in the WRN-depleted WI38hTERT line is driven by a subset of it that is atypical, e.g. is cell cycle-arrested or senesced.

**Figure 7:**
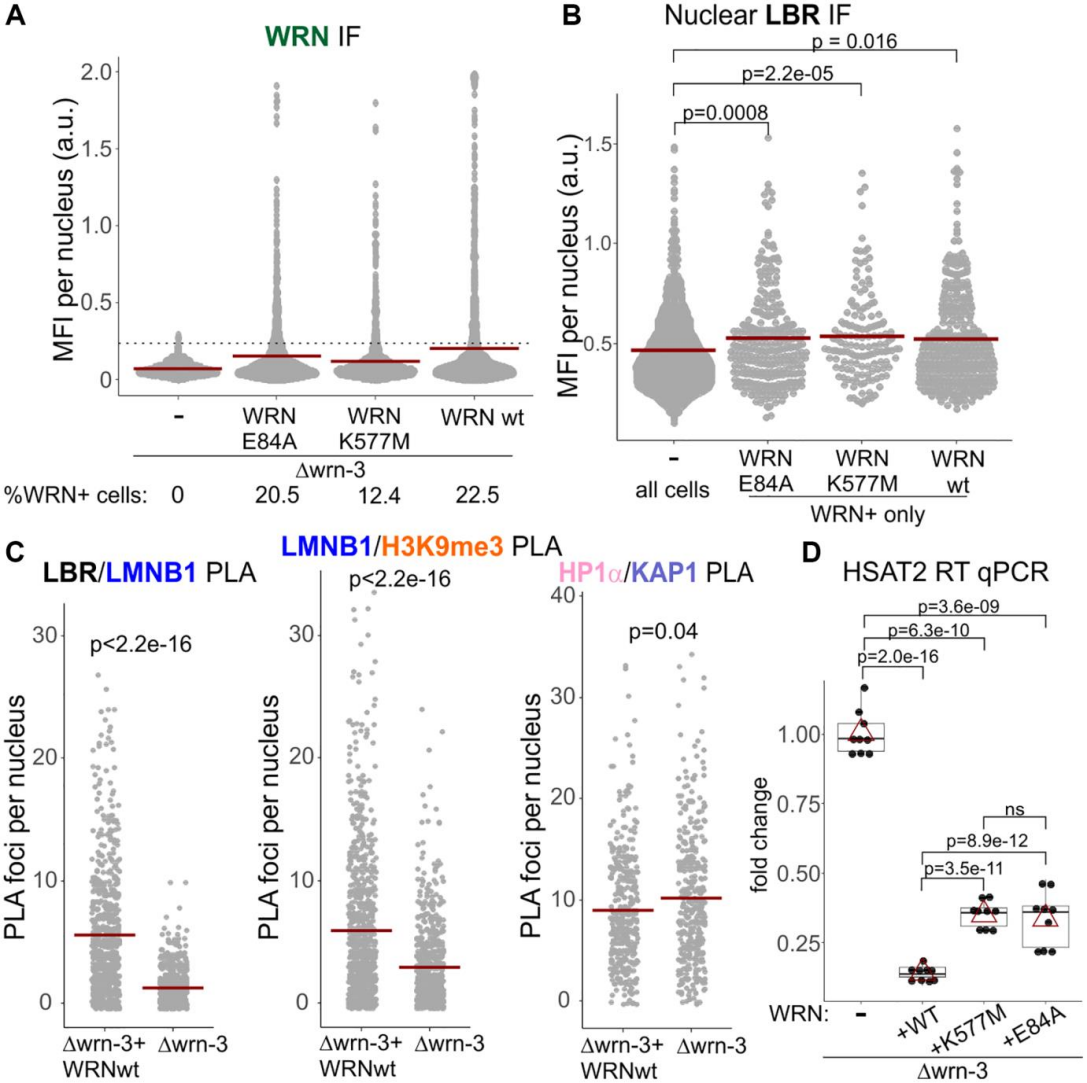
**Catalytically inactive WRN mutants restore nuclear LBR levels and SATII silencing in wrn null cells.** (**A**) Stable expression of the wild type, exonuclease-dead (E84A), or helicase-dead (K577M) WRN in Δwrn-3 cells was measured by IF *in situ* with an antibody against WRN. A dotted line marks the cutoff MFI below which cells were considered negative for WRN expression. (**B**) Nuclear levels of LBR were measured by IF *in situ* in the same cells as in (**A**). For WRN-complemented cells, only WRN-positive cells measuring above the cutoff line in (**A**) for WRN expression were plotted. The graph represents three independent experiments for the wild type and E84A WRN and two independent experiments for the K577M WRN. (**C**) Quantitations of the indicated PLA analyses performed in the Δwrn-3 GM639 with or without expression of the wild type WRN transgene. Crossbars in graphs are distribution means. (**D**) RT-qPCR analyses of SATII satellite repeat transcription performed on Δwrn-3 cells with and without complementation by the wild type or mutant WRN genes. The graph summarizes three independent experiments with technical triplicates. Red triangles are means, and boxplots mark first and fourth quartiles and the distributions’ medians.

Nuclear LBR was also lower in Δwrn-3 GM639 fibroblasts compared to the same cells stably transfected with a lentiviral vector-based WRN transgene (Figure 7). The exonuclease or helicase-dead mutants of WRN, respectively, E84A and K577M, transfected and expressed from the same backbone (Figure 7A and Supplementary Figure 8), were comparable to the wild type WRN in their nuclear LBR levels (Figure 7B). In these experiments, only the LBR fluorescence readouts from WRN-positive cells were included in the measurement since only a subset of cells in these populations expressed detectable levels of WRN (wild type or mutant) and these levels varied (Figure 7A and Supplementary Figure 8). We could not measure WRN levels and PLA signal simultaneously due to an antibody conflict. With that caveat however, we could still observe that re-expression of the wild type WRN in Δwrn-3 cells led to an increase in Lamin B1/LBR and Lamin B1/H3K9me3 PLA signals compared to the uncomplemented Δwrn-3, whereas the HP1α/KAP1 signal was slightly decreased (Figure 7C). Thus, only the Lamin B1 and LBR functional associations and levels were the features consistently reduced by WRN deficiency, whereas HP1α was comparatively less affected.

LBR reduction is sufficient to disrupt CH silencing, including that of pericentromeric satellites [49]. We were able to see SATII RNA derepression in wrn null GM639 compared to the same cells stably transfected with a wild type WRN construct (Figure 7D). Helicase-dead and exonuclease-dead mutants of WRN were both able to suppress SATII expression in the wrn null, albeit not exactly to the same degree as the wild type. This residual difference can be attributed to the above-mentioned lower levels of expression of the mutants. Overall, the data are consistent with a non-catalytic role of WRN in CH silencing via positive regulation of LBR level in the nucleus [49, 50].

**Figure 8:**
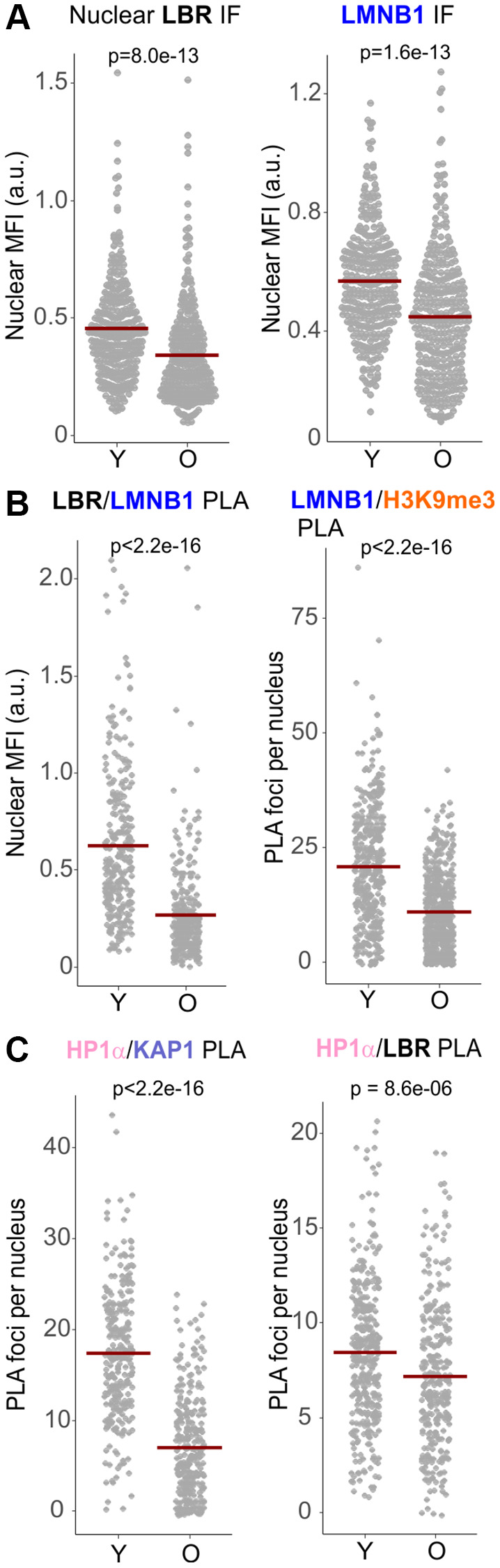
**Replicatively senescing cells reduce the levels of LBR, Lamin B1, and the associated protein complexes.** (**A**) Quantitations of IF *in situ* analyses of the levels of LBR and Lamin B1 in the nuclei of early (young, Y) and late passage (old, O) normal human dermal fibroblasts. (**B**, **C**) Quantitations of the indicated PLA analyses in the same cells as in (**A**). All panels represent two independent experiments. Crossbars in graphs are distribution means.

Reduction of Lamin B1 levels is a prominent feature of senescence [33, 51–54], and at least in some cell types, Lamin B1 or LBR can trigger senescence when depleted [49, 51, 55]. Indeed, both the nuclear levels of Lamin B1 and LBR (by IF *in situ*, Figure 8A) as well as associations of Lamin B1/LBR and Lamin B1/H3K9me3 (by PLA, Figure 8B) were also downregulated in replicative senescing old primary, WRN-proficient fibroblasts compared to the isogenic young fibroblasts. The HP1α/KAP1 PLA signal was also markedly reduced in old vs. young fibroblasts, while the HP1α/LBR signal was reduced to the least degree of all PLA pairs measured (Figure 8C). Nuclear Lamin B1 level was also profoundly reduced in WI38hTERT undergoing OIS, as expected (Supplementary Figure 7C). Interestingly, however, cellular levels of LBR did not appear to reduce in OIS cells, rather, subcellular distribution of LBR changed to a prominently cytoplasmic pattern (Supplementary Figure 7D, 7E). Together, the data indicate that WRN-deficient proliferating cells exhibit an overall milder yet overlapping set of perturbations of the heterochromatin tethering complexes as those seen in WRN-proficient senescing cells.

### LBR reduction in WRN-deficient cells is at least in part mediated through transcription

**Figure 9:**
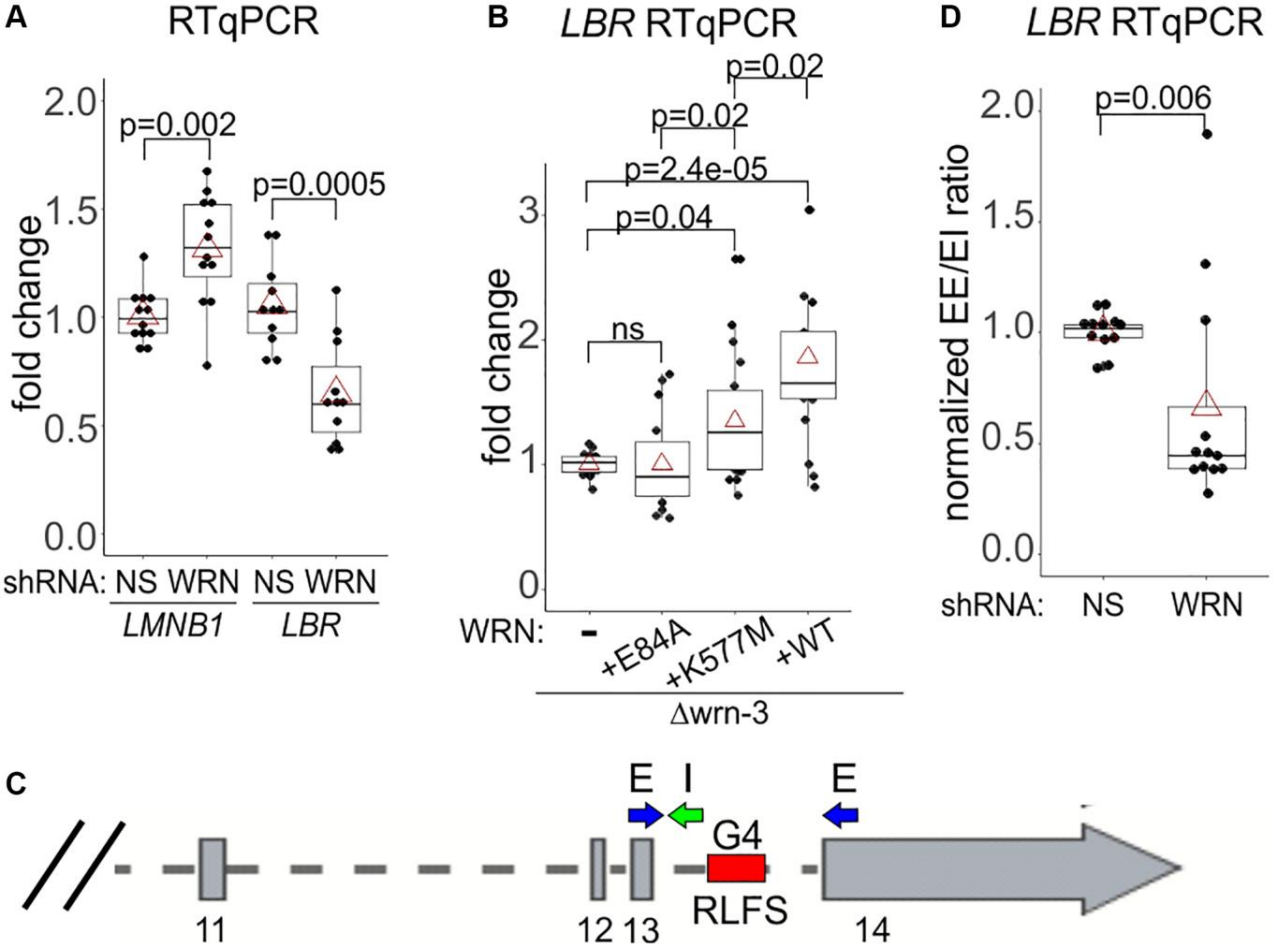
**WRN facilitates transcription of the *LBR* gene.** (**A**, **B**) RT-qPCR measurements of *LMNB1* and *LBR* gene transcripts in the WI38hTERT expressing the indicated shRNAs (**A**) or in Δwrn-3 cells with or without WRN complementation (**B**). (**C**) A schematic of the 3’ end of the *LBR* gene with locations of the qPCR primers and an intronic R-loop forming site (RLFS). Exon numbers are below the gene. (**D**) RT-qPCR measurements of a ratio between the spliced LBR mRNA (EE, no intron 13) and the mRNA containing intron 13 (EI). The data are from WI38hTERT expressing the indicated shRNAs. Graphs in A, B, and D summarize three independent experiments each, with technical triplicates.

The *LBR* transcript was up to 50% reduced in WI38hTERT that were WRN-depleted over a long term (Figure 9A), as well as in the newly-depleted WI38hTERT (Supplementary Figure 5D). On the other hand, the level of the *LMNB1* transcript was not reduced in WRN-depleted WI38hTERT, and in fact, it was slightly elevated upon long-term depletion of WRN (Figure 9A and Supplementary Figure 5D). LBR transcript was also lower in wrn null GM639 cells compared to their wild type WRN-complemented counterparts (Figure 9B). Interestingly, exonuclease-dead mutant of WRN (E84A) was not able to boost LBR transcription despite being expressed at a level closer to the wild type WRN than the helicase-dead mutant, K577M (Figure 7A and Supplementary Figure 8). The K577M mutant, in contrast, is expressed at the lowest level, yet it was able to boost LBR transcription above the background of wrn null cells (Figure 9B). The data are consistent with WRN exonuclease being more important for LBR transcription than WRN helicase.

The *LBR* primer pair that we used maps to exons 13 and 14, the two last exons of the gene, as it is designed to detect a full-length, spliced mRNA (Figure 9C). The approx. 1Kb-long intron 13 between these exons contains an R-loop forming sequence (hg38_RLFS range=chr1:225403587-225404027), recognized as a high confidence RLFS by R-loopBase [56], R-loopDB [57]), and RLBase [58]. Intron 13 RLFS is one of six such sequences in the introns of the *LBR* gene, all of which were experimentally verified to carry R loops *in vivo* as summarized in the above-mentioned databases. Moreover, the non-transcribed strand of the intron 13 RLFS is also rich in G4 motifs (chr1:225,403,870-225,404,030), which are recognized as G4-forming sites by G4Hunter Seeker [59]. We speculated that without WRN these secondary structures may be upregulated in *LBR* intron(s). Conceivably, this may downregulate splicing [60], thus reducing the amount of the spliced mRNA detectable in our qPCR. We repeated RT-qPCRs measuring both the spliced and the intron 13-containing transcripts (Figure 9D). Indeed, the ratio between the levels of qPCR products detecting the spliced (EE in Figure 9) and the unspliced (EI in Figure 9) cDNAs was about two-fold lower in WRN-depleted compared to control WI38hTERT, suggesting that at least the intron 13 of the transcript may be removed less efficiently.

### Replication of SATII is affected by WRN depletion

We next asked whether or not WRN’s effect on heterochromatin maintenance can be explained by WRN’s role in replication. WRN is known to support replication of problematic regions such as fragile sites [5] and inverted repeats [6], and at least some CH may be difficult to replicate due to the repetitive nature of its underlying DNA, which includes inverted repeats [61–63]. This predicts that WRN loss will reduce the rate of progression of forks on CH more dramatically compared to that on average genomic DNA.

**Figure 10:**
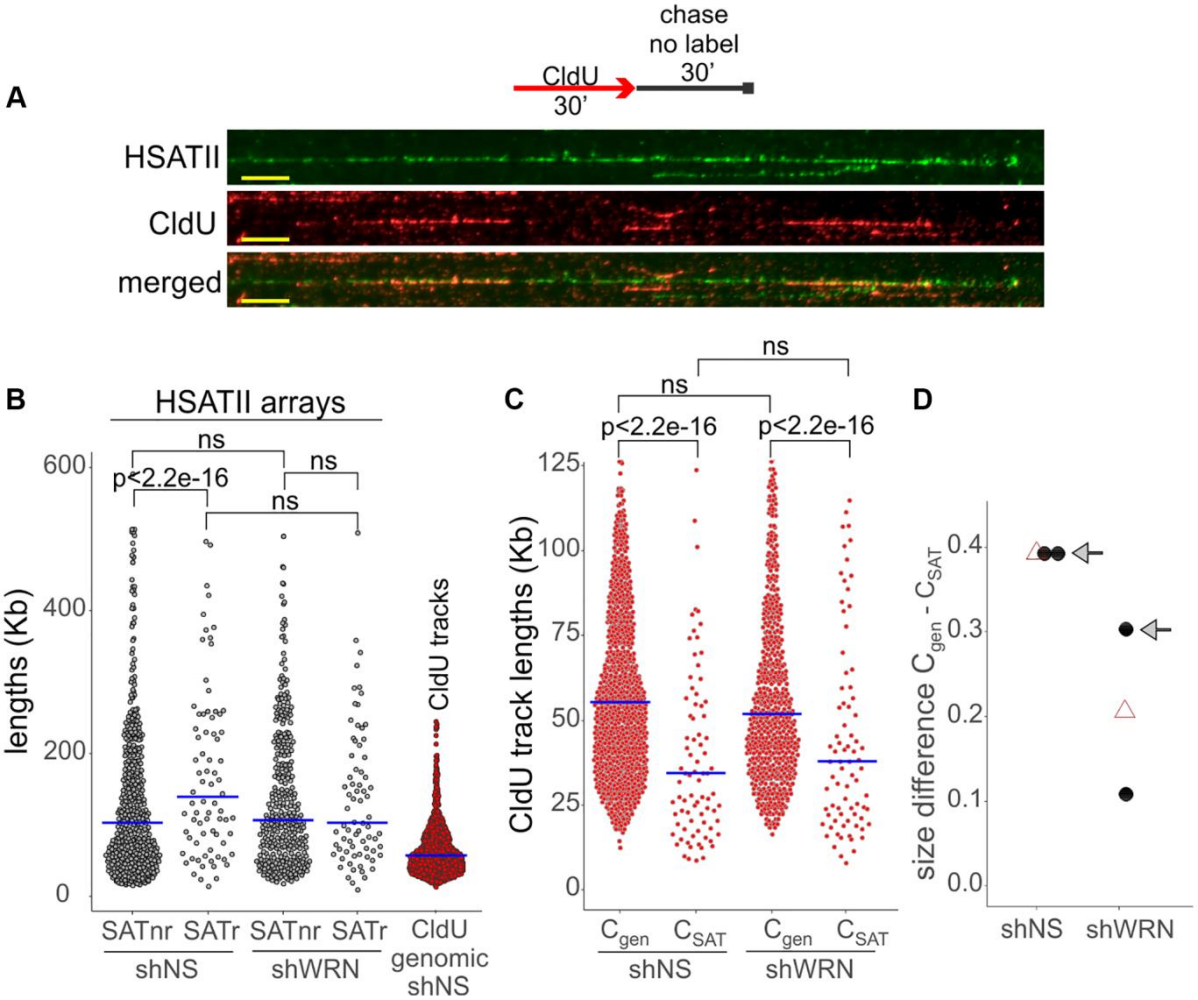
**SATII satellite replication is affected by WRN deficiency in WI38hTERT.** (**A**) A labeling scheme and an example of a HSATII array (green) with CldU replication tracks (red) within it, as visualized by fiber FISH/maRTA. Scale, 10 µm. Cells were labeled with CldU for 30 min and harvested after a 30 min chase in fresh media without label. (**B**) Length distributions of HSATII arrays that do not (non-replicating, nr) or do (replicating, r) carry CldU tracks within them. The length distribution of CldU tracks in total genomic DNA (lane 5), is included for comparison. (**C**) Length distributions of CldU tracks in the genome (C_gen_) and within HSATII arrays (C_SAT_). The plots are representative of two independent experiments. See Methods for the explanation of *p*-value calculations. Crossbars are distribution medians. (**D**) Size of difference between the indicated CldU track length distributions in the control and WRN-depleted cells were determined by deriving Cliff’s delta statistic. Black dots are independent experiments and open triangles are means of the two experiments. The Cliff’s delta values derived from the experiment shown in (**B**) are marked by arrows. Smaller Cliff’s delta values indicate smaller difference between C_gen_ and C_SAT_.

WRN’s effect on replication of any of the satellite repeats has not been studied, and likewise, the replication of SATII class of satellites is not characterized. We examined replication of the SATII satellites using a combination of fiber FISH and maRTA [64], which allowed us to see SATII arrays (green, Figure 10A) as well as replication tracks of CldU incorporation (red, Figure 10A) in stretched DNA fibers. SATII arrays in both control and WRN-depleted cells are on average long enough to encompass a fork progressing for 30 min at an average genomic rate (Figure 10B), however, replication track lengths enclosed within SATII arrays were still dramatically shorter than the global genomic average (compare C_SAT_ and C_GEN_ tracks in Figure 10C), indicating that SATII arrays somehow impede fork progression even in normal cells. In WRN-deficient cells, C_GEN_ tracks were somewhat shorter than in the control, highlighting a subtly reduced global fork progression, as expected [4, 65]. Remarkably, however, replication tracks within SATII arrays, C_SAT_, were longer than the C_SAT_ tracks of the control (Figure 10C). This translated into a reduced size of the difference between the genomic and the SATII-specific fork rates in WRN-depleted cells compared to the control (Figure 10D). These data suggest that, relative to each cell line’s average genomic fork progression, forks progress faster through the SATII arrays in WRN-deficient cells, a result that is contrary to the expected canonical role of WRN in replication described above.

**Figure 11:**
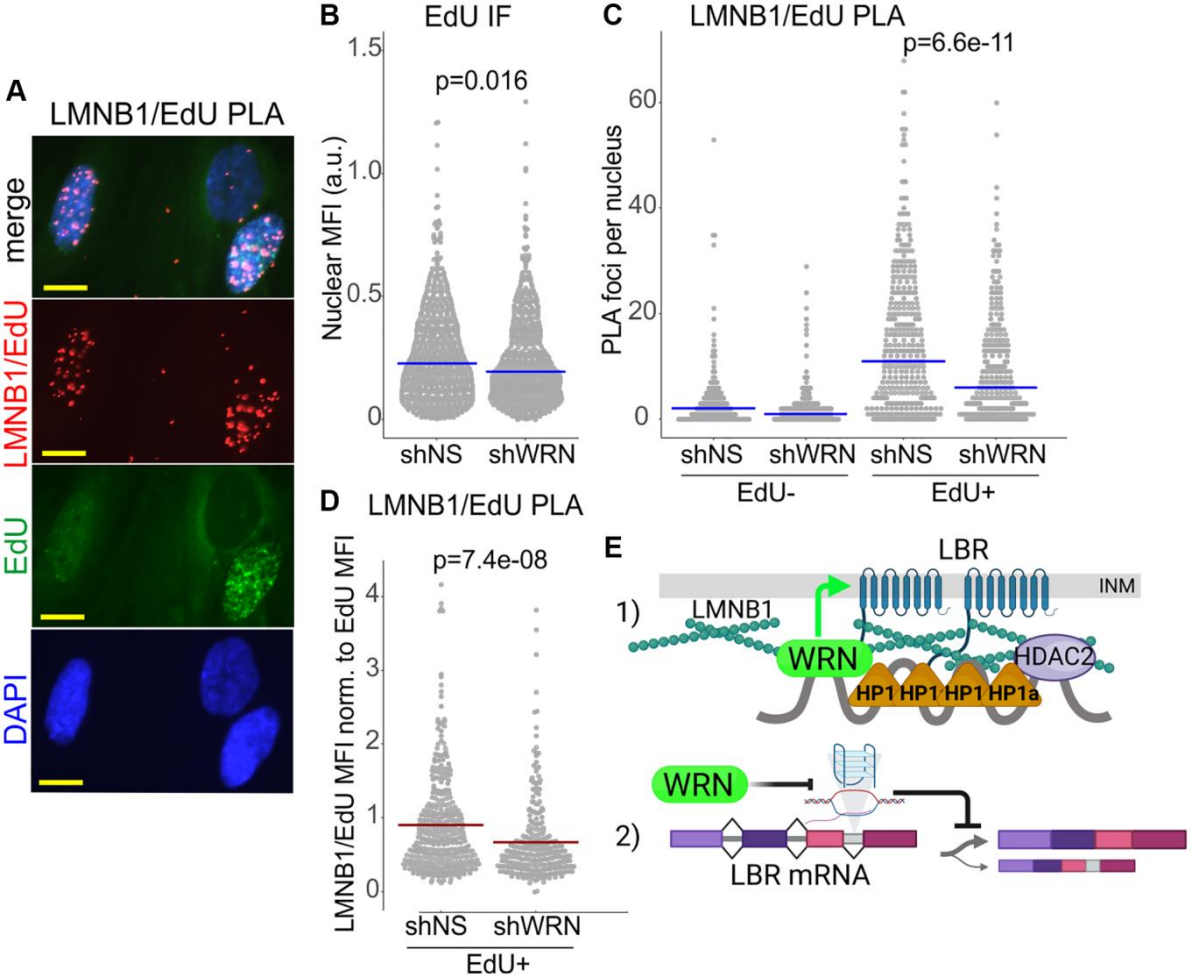
**Lamin B1 association with replicating DNA is reduced in WRN-deficient cells.** (**A**) An example of a PLA analysis detecting Lamin B1 association with nascent DNA *in situ*, demonstrating specificity of the PLA signal to EdU+ positive cells. WI38hTERT cells (expressing non-targeting shRNA) pulse-labeled with 20 µM EdU for 10 min and collected after a 10 min chase. EdU was clicked to a mixture of biotin-azide and Alexa488-azide at a molar ratio of 50:1. PLA was performed with antibodies against Lamin B1 and biotin. Scale bar, 10 µm. (**B**) Quantitation of EdU incorporation in WI38hTERT expressing the indicated shRNAs. (**C**) Quantitation of LMNB1/EdU PLA in the same experiment as in (**B**). The datasets were subsetted into EdU- and EdU+ groups by applying a fixed cut-off EdU MFI value (e.g., 0.02 in (**B**)), below which cells were deemed EdU-. Lamin B1/EdU PLA foci counts were then plotted separately for EdU- and EdU+ subsets. (**D**) For each cell in EdU+ subpopulations, Lamin B1/EdU PLA foci counts were normalized to EdU MFI values and plotted. This normalization compensates for a somewhat lower level of EdU incorporation typically seen in WRN-depleted cells compared to the control (**B**). *P*-values were calculated in Wilcoxon tests. Blue crossbars are medians and red crossbars are means. The results of (**B**–**D**) represent two independent experiments, one of which included biological triplicates. (**E**) A model of WRN roles in CH maintenance. (1) WRN is proximal to HDAC2 via HP1α in the context of Lamin B1/LBR-tethered CH assemblies at the inner nuclear membrane (INM). Reduction of LBR and Lamin B1 in the absence of WRN partially disrupts these assemblies. WRN supports LBR enrichment within the nucleus (green arrow). Possible mechanisms include action on regulators of LBR localization or on nuclear pore complexes. (2) WRN prevents accumulation of R-loops and/or G-quadruplexes in the introns of the LBR gene using its canonical enzymatic activities. Suppression of R-loops and/or G-quadruplexes may promote proper splicing of the LBR transcript.

This paradox can be explained if the rate of replication of SATII arrays is determined by the compact nature of their heterochromatin, as some studies of heterochromatin replication now suggest [66], rather than by the nature of the underlying DNA. According to this view, WRN loss should accelerate fork progression if it causes CH to be more relaxed. To test this, we sought to quantify the WRN-affected CH markers specifically on replicating DNA by using PLA with biotin-conjugated EdU, as previous [26, 67]. Cells were labeled with EdU and collected. The H3K9me3/EdU and LBR/EdU PLA signals were virtually undetectable under those conditions (not shown), however, we were able to detect a robust LMNB1/EdU PLA signal that was specific to EdU-positive cells (Figure 11A). As expected, EdU incorporation in WRN-depleted cells was somewhat lower (Figure 11B). LMNB1/EdU PLA signal was also consistently lower in WRN-depleted cells compared to the control (Figure 11C). This differential could not be due merely to the lower EdU incorporation because it was maintained even after normalization by EdU intensity (Figure 11D). This result is consistent with the notion that CH may be disrupted and thus may be less of an impediment to fork progression over SATII in WRN-depleted cells. In addition, it lends further support to the notion that WRN affects heterochromatin of proliferating cells.

## DISCUSSION

### WRN supports constitutive heterochromatin organization in proliferating cells

WRN-deficiency expedites heterochromatin loss and replicative senescence in mesenchymal stem cells [17]. Here, we asked what phenotypes of heterochromatin loss are attributable directly to WRN status by collecting structural and functional metrics of heterochromatin integrity in proliferating, telomerase-positive human fibroblasts in which WRN was either depleted or deleted, and in isogenic controls. The observed WRN-specific heterochromatin phenotypes were a subset of those seen in senescing fibroblasts. For example, we did not see a global reduction of the heterochromatic histone mark H3K9me3 in proliferating WRN-deficient fibroblasts, suggesting that WRN ablation is not sufficient to cause it. Notably, H3K9me3 loss is not a universal feature of senescence [39].

The HP1α/KAP1 complex, a marker of heterochromatin, was reduced only in the WRN-deficient WI38hTERT but not GM639 background. The apparent absence of this reduction in GM639 may be a detectability issue since not all cells in the complemented cell line express WRN. However, it is also possible that the effect of WRN on HP1α complexes is indirect, and thus is more buffered against WRN reduction. This is also consistent with the fact that both in WI38hTERT and GM639 WRN absence had no impact on association of HP1α with HDAC2. Further dissection of these dependencies will require identifying WRN mutants defective in specific associations.

In contrast, WRN deficiency consistently reduced Lamin B1 and LBR levels and their functionally important associations with each other and with chromatin. Lamin B1 and LBR are responsible for tethering and compacting heterochromatin at the inner nuclear membrane, both via the HP1α protein and by directly interacting with other chromatin components [68, 69], and their ablation is sufficient to disrupt CH silencing [49, 50].

Importantly, LBR and Lamin B1 downregulation is observed in senesced cells [33, 34, 41, 70–72] and in some cell types, it is in fact sufficient to trigger senescence or SASP [49, 51, 55, 73]. Furthermore, LBR downregulation was shown to cause reduction of Lamin B1 levels [74]. Thus, the reduction of Lamin B1 level in WRN deficiency may be explained by the reduced level of LBR.

Though we were not able to see physical proximity of WRN to LBR or Lamin B1 with the antibodies used in this study, our findings of HP1α, HDAC2, and WRN associations agree with prior research [17] in indicating that WRN regulates LBR while also being a physical constituent of the protein assemblies of heterochromatin. We envision that WRN, HDAC2, and HP1α are proximal within the context of compacted heterochromatin. Their mutual proximity is dependent on heterochromatin tethering to LBR and Lamin B1 (Figure 11E) and can be downregulated by disruption of Lamin B1 or LBR levels or distribution, including at the onset of senescence. In its turn, WRN loss may cooperate with senescence triggers by partially disrupting CH, and thereby accelerating the onset of senescence.

### Canonical and non-canonical functions of WRN contribute to heterochromatin maintenance

One goal of our study was to determine if CH maintenance by WRN could be traced to the known functions of WRN or represented a novel function. Our data suggest two ways by which WRN loss can compromise Lamin B1 and/or LBR-containing assemblies, and these two ways differ in their dependence on WRN enzymatic activities. First, we detected an approximately two-fold reduction of the LBR transcript in WRN-deficient compared to WRN-proficient cells. These data align with and confirm the LBR data in the transcriptomic datasets generated for isogenic WRN-depleted and control NHDFs [75] and in WRN KO vs. control MSCs [17]. Two studies [75, 76] noted that many of the genes whose transcription is affected by WRN contain potential G4-forming sequences at TSS and in introns. The authors proposed that WRN controls G-quadruplex levels at these genes, albeit there currently is no general consensus as to whether loss of WRN results in up- or down-regulation of such genes. Our data invited a hypothesis that WRN suppresses R-loops and/or G-quadruplexes in the *LBR* gene introns. This function should require at least one of WRN’s catalytic activities [8–12]. In turn, upregulation of these secondary structures in WRN’s absence may negatively affect splicing of the *LBR* transcript. As such, this mechanism is distinct from the recently uncovered suppression of the LBR transcript by the micro-RNA miR-340-5p that is induced in senescing WI38 cells [49]. Indeed, transcriptomic data did not show elevated miR-340-5p in WRN-deficient fibroblasts [75]. Importantly, if translated, the unspliced intron 13 of LBR mRNA will terminate the LBR ORF prematurely at amino acid 521, and a similar truncation (at aa534) is known to cause Greenberg skeletal dysplasia and was shown to result in displacement of LBR into nucleoplasm and its proteasome-dependent degradation [77]. This, together with the possibility that the intron 13-containing mRNA may undergo nonsense-mediated decay, provides a plausible mechanism for reduction of total LBR in WRN-deficient cells via loss of the canonical function of WRN in suppressing secondary structures in DNA. However, given that additional RLFS and non-overlapping G-quadruplex sites are found throughout the LBR gene, secondary structure-mediated disruptions to more than one step of LBR transcript expression cannot be ruled out. Likewise, it remains to be determined whether an R-loop or a G-quadruplex is the critical structure responsible for the WRN-dependency of LBR transcription. However, the fact that WRN exonuclease-dead mutant was very similar to wrn null and more defective than WRN helicase-dead mutant for LBR transcription, and WRN exonuclease acts on R-loops but not G-quadruplexes [10–12] points towards R-loops as a primary culprit.

WRN loss also reduced enrichment of the LBR protein in the nucleus, an effect that did not require WRN helicase or exonuclease activities, and may point to a novel, noncanonical function of WRN. Subcellular distribution of LBR is dynamically regulated through cleavage, turnover and post-translational modification [78–81]. Phosphorylation of the arginine and serine-rich (RS) domain of LBR by the SRPK1 kinase leads to a more cytoplasmic distribution of the protein [79]. Hyperphosphorylation of the RS domain may disrupt oligomerization of LBR [47], which can be a factor in its retention at INM as well as in facilitating compaction of LBR-tethered heterochromatin. This mechanism can plausibly require WRN protein-protein interactions but not its enzymatic activity. Another possible mechanism can be derived from the earlier studies where WRN, via its interactor WHIP1, was found associated with the nuclear pore complex, NPC, proteins [82] that also promote LBR localization to the INM [79, 83, 84]. Thus, it is possible that WRN can affect LBR localization by modulating NPC activity or density *in cis* by a yet unknown mechanism.

In our effort to trace WRN CH phenotypes to its known functions, we also examined satellite repeat replication. Supporting progression of forks globally in the genome and specifically in the difficult-to-replicate regions such as fragile sites is a known, canonical function of WRN [4, 5, 65]. Given that satellite repeats are viewed as potentially difficult to replicate and indeed display a significantly slower fork progression rate compared to the genomic average ([85] and Figure 10), we anticipated that WRN loss would have a disproportionately large slowing effect on fork progression on SATII arrays. Surprisingly, if anything, WRN absence accelerated forks on a relative scale. It is possible that the protein complexes in charge of maintaining satellite repeat replication specifically exclude WRN. However, given the evidence of physical presence of WRN at SATII [17], this possibility seems less likely. Instead, we propose that a compact nature of CH is the factor that reduces the rate of fork progression on SATII. A negative impact of heterochromatin on replication fork rate is supported by the findings in which global loss [86] or global gain of heterochromatin [87, 88] caused, respectively, fork acceleration or fork slowing/stalling. Multiple findings of our study support the conclusion that CH is perturbed in WRN-deficient cells, providing a plausible cause for a relatively fast fork progression on SATII in WRN-deficient cells.

## CONCLUSION

Our study highlights WRN as a contributor to the integrity of CH and points at the altered levels and distribution of LBR as a mediating mechanism. Our demonstration of this role of WRN in immortalized, telomerase-positive, replicating cells rules out that these effects are mediated or influenced by the regulatory circuits that induce senescence. Our study has also established several PLA markers of senescent state, which can be used in senescence and aging studies.

LBR abundance in the cell is controlled at the protein and RNA levels by multiple mechanisms [49, 78, 80, 89, 90], evidencing its importance but also the complexity of its regulatory network; and our data highlight WRN as one more member of this network. The results also raise a question of whether the differential utilization of LBR as a heterochromatin tether by different cell types [80, 89, 91] can help to explain cell lineage-specific differentials in the severity of WRN deficiency, a phenomenon that is referenced in a definition of the Werner syndrome as a segmental progeria [92, 93]. During iPS cells differentiation into mesoderm, LBR distribution changes from the predominantly nuclear to the mixed nuclear/cytoplasmic pattern [90], which potentially reduces the effective nuclear concentration of LBR. Incidentally, it is the mesenchymal lineage that is most markedly affected by WRN loss [93]. It is tempting to speculate that lineages that are either more dependent on LBR and/or more rate-limiting for LBR in the nucleus may be more severely impaired by WRN loss. In this light, it will be of future interest to determine if upholding high levels of LBR may alleviate the negative effects of WRN in MSCs, though it is also possible that high LBR will perturb MSC differentiation. Further research is necessary to test this hypothesis and to fully understand the details of the functional interaction between WRN and LBR.

## MATERIALS AND METHODS

### Cells and culture

The telomerase-positive, SV40-transformed human dermal fibroblast GM639 (GM00639, Cellosaurus ID CVCL_7299) and its derivatives have been used by us previously [26, 94, 95] and were originally obtained from the NIGMS Human Genetic Mutant Cell Repository. Early and late passage normal human dermal fibroblasts (NHDFs) were generated in-house and are described in [96]. GM639 and NHDFs were grown in high glucose Dulbecco’s Modified Minimal Essential Medium (DMEM) with L-glutamine, 10% fetal bovine serum, FBS, (Hyclone) and antibiotics. The immortalized WI38hTERT fibroblast line was a gift of Dr. Carl Mann and contains an inducible GFP-RAF1-ER transgene [43]. WI38hTERT was grown in Minimal Essential Medium (MEM) with L-glutamine and sodium pyruvate (Gibco) supplemented with 10% FBS, Nonessential Amino Acids, and antibiotics. All cell lines were kept in a humidified 5% CO_2_, 37°C incubator. Mycoplasma testing was performed using the UW/FHCC Cancer Consortium Shared Resource Specimen processing service https://www.fredhutch.org/en/research/shared-resources/core-facilities.html.

### Drugs and other reagents

Stock of 5-chlorodeoxyuridine (CldU, Sigma-Aldrich) was at 10 mM in PBS, and 5-ethynyldeoxyuridine (EdU, Sigma-Aldrich or Click Chemistry Tools) was at 10 mM in DMSO. CldU was used at a concentration of 100 µM and EdU was used at 10 or 20 µM. Doxycycline stock solution was at 100 µg/ml M in PBS and it was used at 100 ng/ml final concentration. 4-hydroxytamoxifen, 4-HT, stock (Sigma-Aldrich) was 40 µM in ethanol and was used at a final concentration of 20 nM. All stocks were stored at −20°C.

### Constructs and CRISPR-Cas9-mediated gene knockout

gRNAs were designed against the *WRN* gene sequences GGTAATATTTAACCTCCGT and GTCTATCCGCTGTAGCAAT. The two gRNAs were cloned into the pLenti-L3US2-RFPv3 vector, and the procedures were as described before [25, 26]. Briefly, GM639 cells were transduced with a lentiviral vector pLenti-Cas9-2TA-emGFP, and flow-sorted for positives. These cells, GM639-Cas9, were further transduced with dgRNA-expressing pLenti-L3US2-RFPv3. Transduced mass cultures were sorted for RFP-positive cells, and after expansion, the loss of expression of WRN was verified by Western blotting. Individual clones were subsequently derived from this cell culture, Western blot verified, and the regions surrounding gRNA sites were PCR-amplified and sequenced.

Virus generation and cell transduction were as described [94].

WRN expression constructs pLX209-neo-WRN, pLX209-neo-WRN E84A, pLX209-neo-WRN K577M were a gift from Francisca Vazquez (respectively, Addgene plasmids #125788, http://n2t.net/addgene:125788; RRID: Addgene_125788; #125789, http://n2t.net/addgene:125789; RRID: Addgene_125789 and #125790; http://n2t.net/addgene:125790; RRID: Addgene_125790) [97].

pLKO.1-Puro-shWRN2 TET ON and pLKO.1-Puro-shNS TET ON for doxycycline-inducible shRNA expression were a gift from Dr. Weiliang Tang. shWRN2 is identical to shWRN2-4 described in [94] and the non-targeting shNS is derived from that described in [75].

### SA-βgal staining

SA-βgal staining was done using SPIDER-βgal senescence detection kit (Dojindo) according to the manufacturer’s recommendations for fixed cells.

### RNAi-mediated depletion

siRNAs against HP1α (CBX5) Hs_CBX5_5, Cat. No. SI03146479 and All Stars negative control SI03650318 siRNAs were from Qiagen. siRNA was transfected with lipofectamine RNAiMAX (Invitrogen) according to the manufacturer’s protocol. Experiments were performed 36 to 48 hrs post-transfection with individual siRNAs. Depletion was verified in each transfection by Western blotting.

### Antibodies

Antibodies were as follows: rabbit α-biotin Cat. No. A150-109A (Bethyl); mouse α-BrdU/CldU Cat. No. NBP2-44055 (Novus); rabbit α-STING Cat. No. 19851-1-AP (Proteintech), mouse α-NCL Cat. No. 396400 (Life Technologies), mouse α-Lamin A/C Cat. No. sc-376248 (Santa Cruz Biotechnology), rabbit α-GAPDH Cat. No. 5174 (CST), rabbit α-Histone H3K9me3 Cat. No. 13969 (CST), rabbit α-Histone H3K9me3 Cat. No. A2217P (Diagenode), mouse α-WRN, Cat. No. W0393 (Millipore-Sigma), rabbit α-WRN, Cat. No. NB100-471 (Novus); mouse α-HP1α Cat. No. NBP2-52420 (Novus), rabbit α-HP1α Cat. No. 2616 (CST); rabbit α-V5 Cat. No. 3202 (CST); rabbit α-KAP1 Cat. No. 4124 (CST); mouse α-HDAC2 Cat. No. 5113 (CST); rabbit α-HDAC2 Cat. No. 57156 (CST); rabbit α-LBR Cat. No. A5468 (ABclonal); mouse α-Lamin B1 Cat. No. 66095-1 (Proteintech).

### Western blotting

Proteins were visualized on Western blots by ECL (Thermo Fisher Scientific) and quantified using FluorChem Imager (Alpha Inotech). For presentation, images were saved in TIFF format, adjusted for brightness/contrast and cropped in GIMP, then assembled into figures in CorelDraw. Image brightness/contrast adjustments were made across all lanes of each protein measured. In some cases, lane order was changed and extra lanes were deleted.

### Chromatin immunoprecipitation (ChIP)

ChIP was performed according to the Abcam protocol as described in [95], with 5 µg of chromatin using rabbit α-Histone H3K9me3 Cat. No. A2217P (Diagenode) or the equivalent amount of rabbit IgG Cat. No. C15410206 (Diagenode), and α-histone H3 Cat. No. 4499S (CST), rabbit α-LBR Cat. No. 12398-1-AP (Proteintech), or the equivalent amounts of rabbit IgG Cat. No. 2729S (CST). Input and pulldown DNA was analyzed in triplicates by qPCR with iTaq Universal SYBR Green supermix (Bio-Rad) and the following pairs of primers:

LINE1 5′UTR: 5′GATGATGGTGATGTACAGATGGG3′ and 5′AGCCTAACTGGGAGGCACCC3′ [98]. SATII: 5′TCATCGAATGGAAATGAAAGGAGTCATCATCT3′ and 5′CGACCATTGGATGATTGCAGTCAA3′ [95]; αSAT (CST Cat. No. 4486). Only the experiments in which IgG pulldowns were uniformly low (less than 1/10 of the value) were used for further analysis for H3K9me3 and HH3. T1LAD-1: 5′CCTTGAAGAGGTCCTTCACATC3′ and 5′TTGAGTAGGAGTGGTGAGAGAG3′ [48]; NonLAD-1: 5′GCTGTATCTTGGAGGCTATCAC3′ and 5′CCTCCCAAAGTGCTGGAATTA3′ [48].

### RNA isolation and RT-qPCR

RNAs were isolated using RNeasy Plus RNA isolation kit (Qiagen) and additionally purified of DNA contamination using DNA-free kit (Thermo Fisher Scientific). 2 µg of RNA was reverse-transcribed using High-Capacity cDNA Reverse Transcription kit (Applied Biosystems) per manufacturer’s protocol, with GAPDH-reverse primer 5′GATGCAGGGATGATGTTCTG3′ and HSATII primer 5′ATCGAATGGAAATGAAAGGAGTCA3′. No RT controls were performed for every sample. cDNAs were diluted 1:2 and 1 µl of diluted cDNA was used per qPCR reaction. Reactions were run in triplicates with iTaq Universal SYBR Green supermix (Bio-Rad) and the following pairs of primers: 5′ACAACTTTGGCATTGAA3′ and 5′GATGCAGGGATGATGTTCTG3′ for GAPDH; 5′TCATCGAATGGAAATGAAAGGAGTCATCATCT3′ and 5′CGACCATTGGATGATTGCAGTCAA3′ for HSATII. After verifying that no-RT negative control reactions displayed higher Ct values than +RT reactions, the data were processed as follows. Triplicate Ct values for GAPDH were averaged and subtracted from HSAT values to derive ΔCt values, which in turn were used to derive ΔΔCt values, for differences between samples within each experiment, and the fold change values (as 2^−ΔΔCt^). Significance was determined in paired two-tailed *t*-tests performed on ΔΔCt values.

For LMNB1 and LBR RT-qPCR, the procedures were as above except that RT reactions were performed with random primers. *LMNB1* and *LBR* (exonic) primers used are described in [99], and the intronic LBR primer is 5′CACACATCTTTCTGGCGG3′.

### Proximity ligation assay (PLA) and immunofluorescence (IF) *in situ*

For PLA detection of protein proximity to nascent DNA, cells were labeled with 20 µM EdU (Click Chemistry Tools) for 20 min and EdU was clicked to a mixture of Alexa488 and biotin azides at a molar ratio of 1 to 50. Protein/protein PLA was performed according to the standard procedures. PLA DuoLink red or green detection reagents and DuoLink anti-mouse and anti-rabbit antibodies (Millipore-Sigma Cat. No DUO92008, DUO92014, DUO92001, and DUO92002, respectively) were used as described previously [67], except that after formaldehyde fixation cells were washed in PBS, permeabilized by the addition of 4°C 90% methanol in PBS and stored at −20°C prior to staining. The staining was preceded by two washes in PBS, an incubation with 0.25% triton X100 for 10 min, and two more washes in PBS. IF *in situ* was performed as described before [26] except in the case of KAP1 IF, which was performed according to the manufacturer’s protocol. Images of cells were collected under Zeiss Axiovert 200M microscope with 40X magnification objective using Micro Manager software. Digital images were analyzed with Fiji ImageJ Software package with custom macros as described in [67] or with the Cell Profiler software package.

### Fiber FISH and microfluidics-assisted replication track analysis (maRTA)

DNA stretching was performed as described before [25, 64]. After drying at room temperature overnight, coverslips were incubated in 2XSSC, 70% formamide at 75°C for 5 min, dehydrated in 70, 85, 95, and 100% ice-cold ethanol series for 2 min each, and air-dried. Hydridization mixture containing 50% formamide, 20 mM Tris HCl pH7.4, 5 mg/ml blocking reagent (Roche), and 0.5 µg/ml biotinylated LNA probe against SATII (based on the sequence ATTCCATTCAGATTCCATTCGATC [40] and custom-produced by IDT) was incubated at 80°C for 5 min and aliquoted onto a glass slide prewarmed to 80°C. Coverslips were placed DNA side down into hybridization mixture droplets, incubated at 80°C for 5 min and then placed into a humidified chamber at room temperature for 2 hrs. Coverslips were then washed in 2× SSC, 0.1% Tween 20, once at room temperature and once at 50°C, for 10 min each. Coverslips were rinsed in maRTA antibody wash buffer, then blocked and stained with rabbit α-biotin Cat. No. A150-109A (Bethyl) and mouse α-BrdU/CldU Cat. No. NBP2-44055 (Novus) antibodies followed by secondary antibodies as in [64]. Microscopy of stretched DNAs was performed on the Zeiss Axiovert microscope with a 40× objective as above. Lengths of tracks were measured in raw merged images using Zeiss AxioVision software. Fluorochromes were Alexa594 for CldU and Alexa488 for biotin.

### Statistical analysis

Statistical analyses and graphing of the data were done in R studio. *P*-values for qPCR results were derived from pairwise *t*-tests on ΔΔCq values, with Benjamini-Hochberg correction for multiple comparisons. *P*-values for the rest of the assays were as follows. For continuous variables (i.e., track lengths, mean fluorescence intensities) *p*-values were calculated in K.S. tests and for discrete variables (i.e., PLA foci) – in Wilcoxon tests. The analyses were always performed on whole datasets, without exclusion of any outlier data points. To quantify and concisely visualize the sizes of differences between PLA foci, track lengths, or MFI distributions of pairs of samples, we calculated the Cliff’s delta statistic for these pairs of samples. Cliff’s delta is a metric recommended for comparison of non-parametric distributions. In general, Cliff’s delta of a distribution A vs. distribution B can range from 1 (if all values in A are larger than all values in B) to −1 (if the reverse is true), and 0 value indicates that the distributions are completely overlapping. For fiber FISH/maRTA, two types of *p*-values were derived. When comparing independent samples, i.e., SATII array lengths in control and WRN-depleted cells, *p*-values were derived in K.S. tests. When comparing samples in which one sample was a subset of another, for example, lengths of CldU tracks within SATII arrays (C_SAT_) vs. all genomic CldU tracks (C_gen_), *p*-values are probabilities with which a subset the size of *n* = n of C_SAT_ and a mean value equal to that of C_SAT_ can be randomly drawn from all CldU tracks (C_gen_+ C_SAT_). To calculate it, random subsets of *n* = n of C_SAT_ were drawn 100 times from a dataset of all CldU tracks, a mean of each subset was calculated, and a two-sided one-sample *t*-test was performed on a resulting distribution of means for the null hypothesis that its mean equals the mean of C_SAT_ at a confidence level = 0.98.

## Supplementary Materials

Supplementary Figures
