# Supplementary material for: Werner syndrome RECQ helicase participates in and directs maintenance of the protein complexes of constitutive heterochromatin in proliferating human cells
Source: Aging (Albany NY). 2024 Oct 17;16(20):12977–3011. doi: 10.18632/aging.206132 (PMC11552638; doi:10.18632/aging.206132)
Supplement: Supplementary Figures [file aging-16-206132-s001.pdf]

SUPPLEMENTARY FIGURES

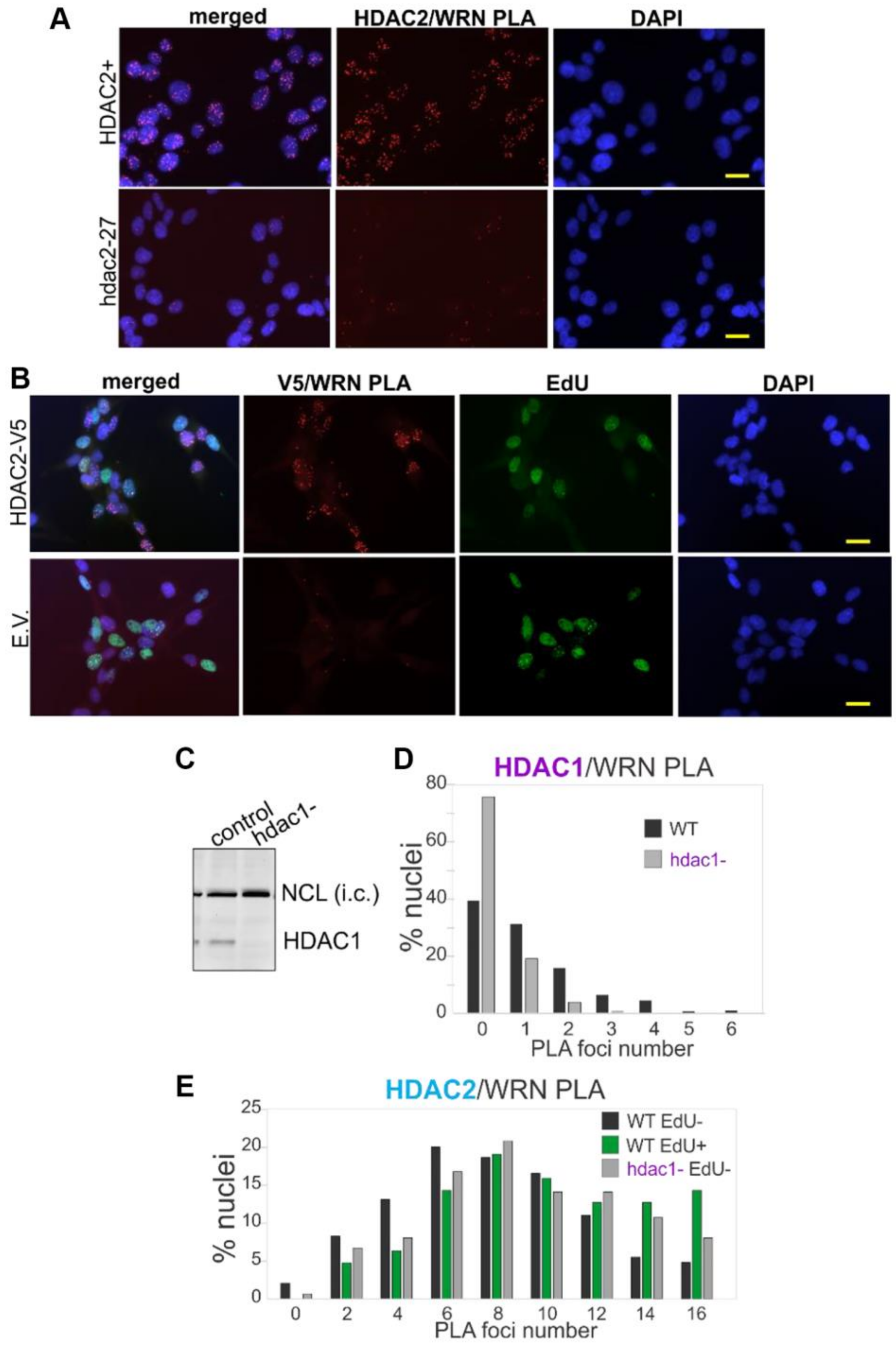

Supplementary Figure 1. Proximity of WRN and HDAC2 is not dependent on HDAC1 and not limited to S phase cells. (**A**, **B**) Examples of HDAC2/WRN and V5/WRN PLA results. Note that the images in (**A**, **B**) are zoomed-out and split-channel versions of the images shown, respectively, in Figure 1A and 1E. In (**B**), E.V., empty vector, and HDAC2-V5 are the constructs stably expressed in hdac2-27 SV40-immortalized fibroblasts. Cells were labeled with 20 µM EdU for 30 min before fixation. Scale bar, 20 µm. (**C**) A Western blot demonstrating a clonally-derived knockout of *HDAC1* in normal dermal human fibroblasts (NHDFs). (**D**, **E**) Quantitations of HDAC1/WRN (**D**) or HDAC2/WRN (**E**) PLA analyses in the control (wt) and *hdac1* null NHDFs. The wild type cells were labeled with 20 µM EdU for 30 min before fixation, and the incorporated EdU was clicked to Alexa488 azide. HDAC2/WRN PLA foci distributions were plotted separately for EdU- and EdU+ cells.

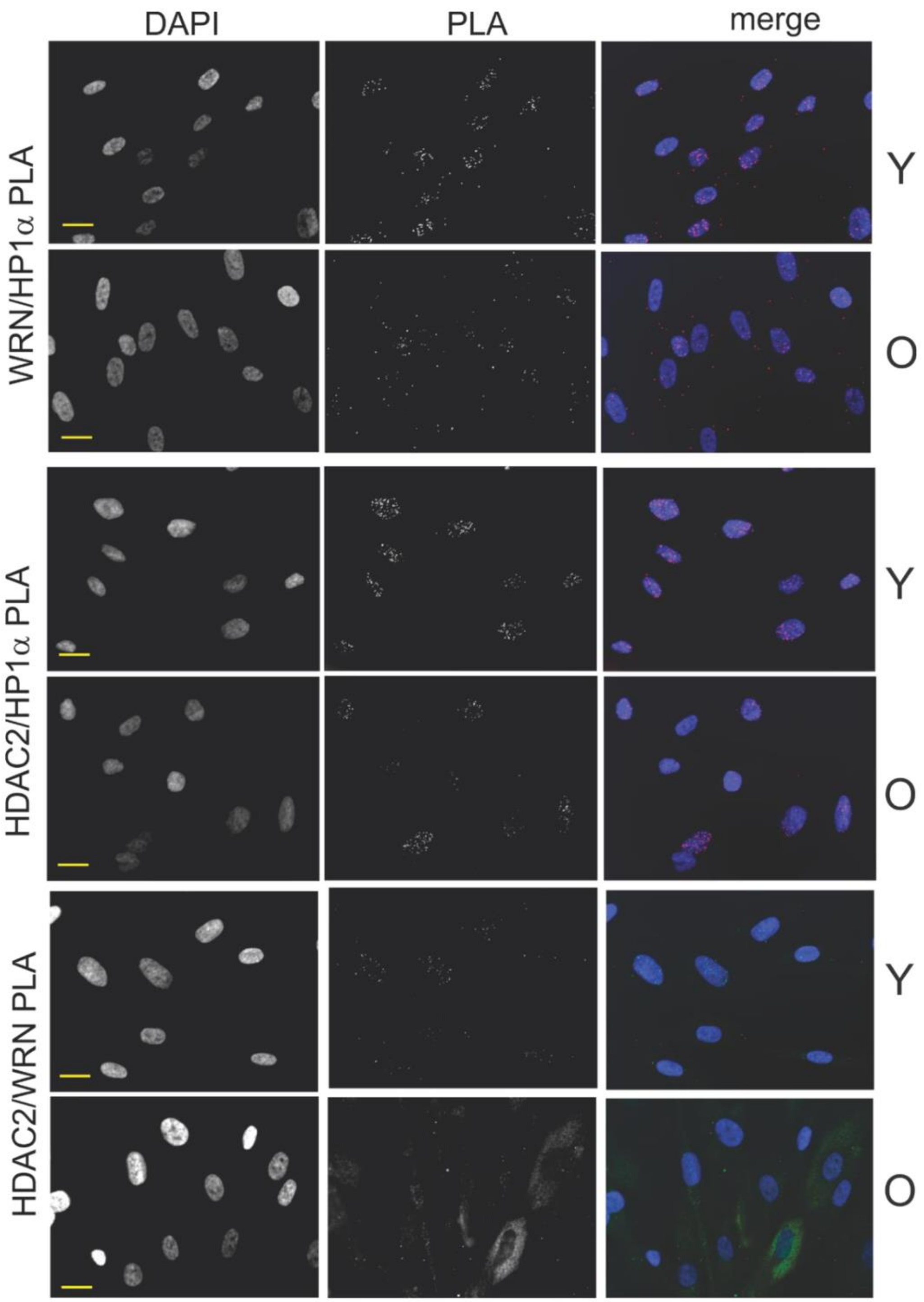

Supplementary Figure 2. Examples of PLA analyses involving WRN, HDAC2, and HP1α in early (Y) and late (O) passage normal human dermal fibroblasts. Scale bar, 20 μm.

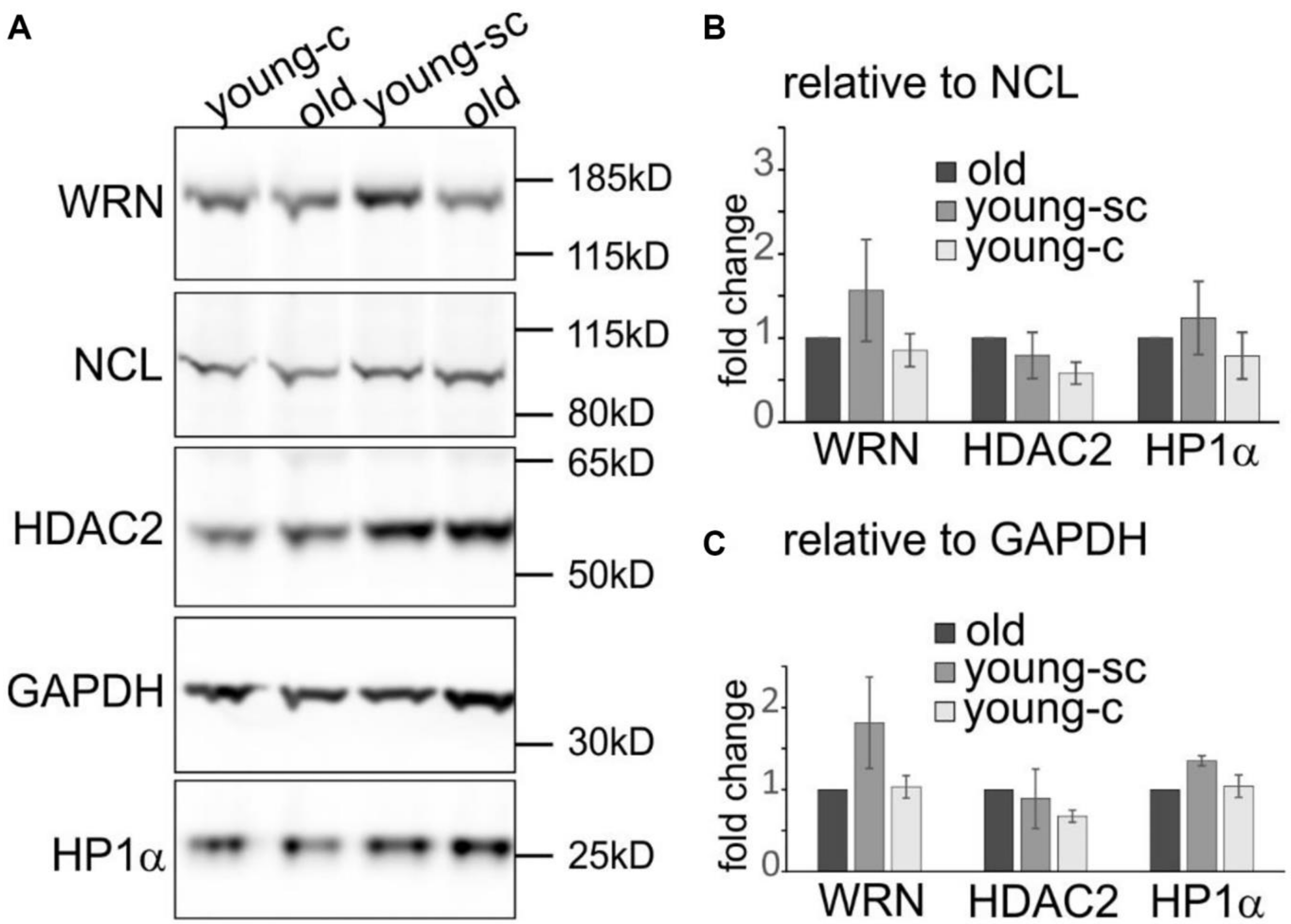

Supplementary Figure 3. WRN, HDAC2, and HP1α levels in late-passage versus early-passage normal human dermal fibroblasts (NHDFs). (**A**) A Western blot showing the levels of the indicated proteins in early-passage, confluent (young-c), sub-confluent, proliferating (young-sc), and late-passage, senescing (old) NHDFs. (**B**, **C**) Data from two to four independent experiments as in (**A**) were quantified and plotted. NCL (**B**) or GAPDH (**C**) loading controls were used for normalization. Data are presented relative to each protein's level in old NHDFs.

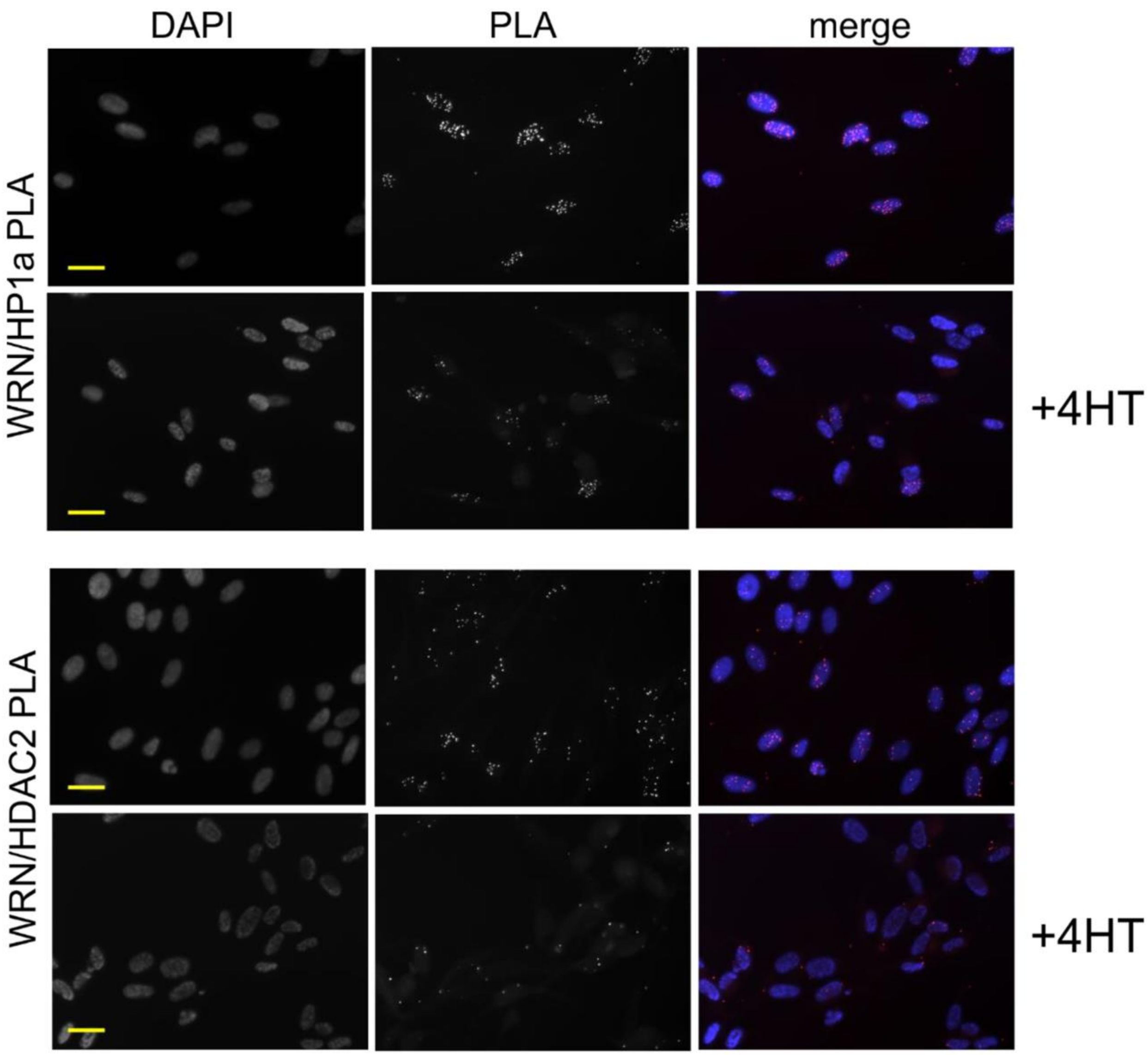

Supplementary Figure 4. Examples of PLA analyses involving WRN, HDAC2, and HP1α in proliferating and RAF oncogene-senesced (+4HT) WI38hTERT. Scale bar, 20 µm.

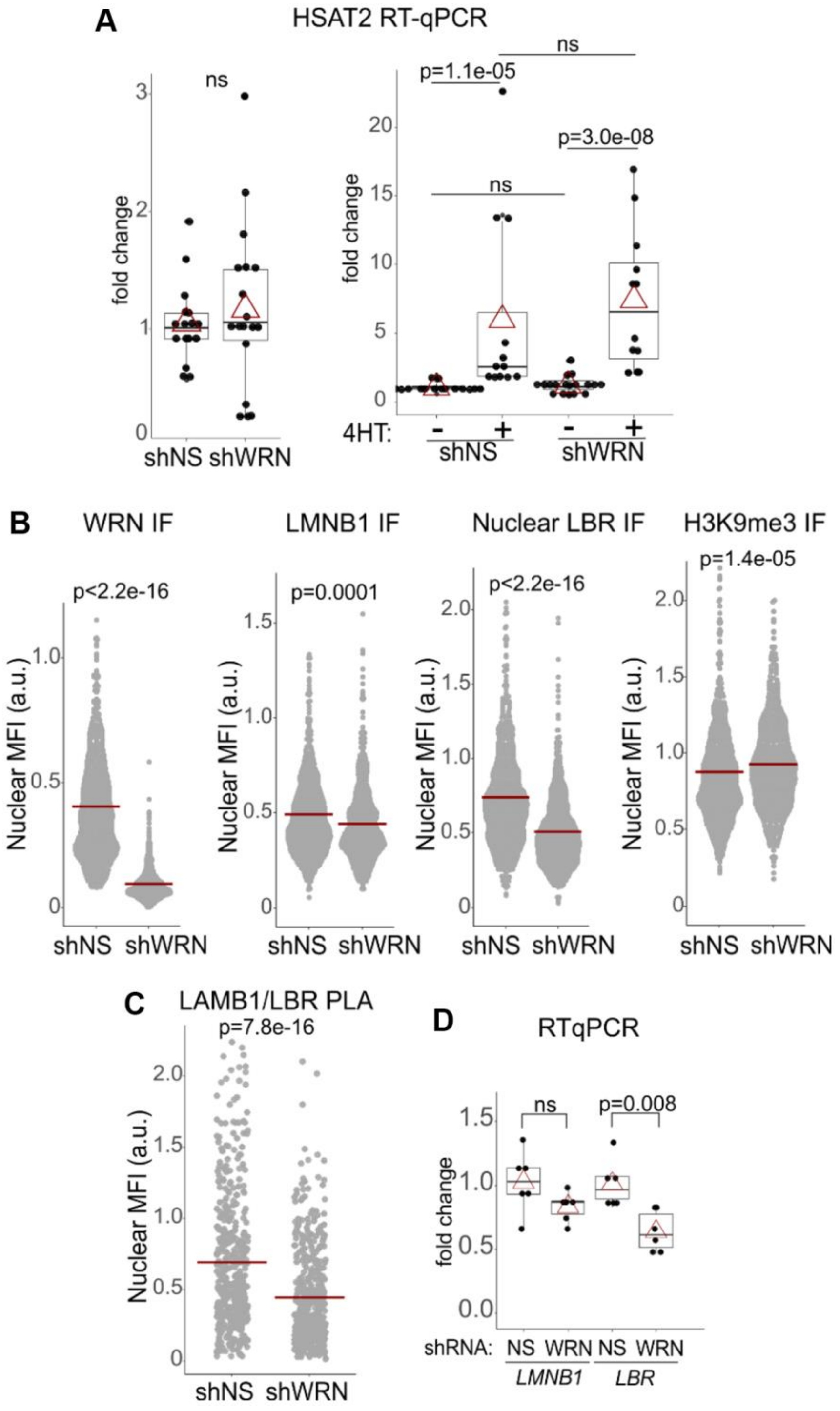

Supplementary Figure 5. (**A**) RT-qPCR analyses of SATII transcript levels in senescing (+4HT) or proliferating (no 4HT) WI38hTERT expressing the indicated shRNAs. No-4HT samples in the left and right panels are the same but are shown at different resolutions of the Y-scale. The graphs summarize six independent experiments with technical triplicates. *P*-values were determined in paired *t*-tests with Benjamini-Hochberg correction for multiple comparisons. (**B**) Quantitations of the indicated IF *in situ* analyses in the control and WRN-depleted WI38hTERT with stably integrated TET-inducible shRNA constructs (shNS or shWRN) that were treated with 100 ng/ml doxycycline for five days before collecting the cells for the indicated analyses. (**C**) Quantitation of LMNB1/LBR PLA analysis in the same cells as in (**B**). *P*-values in (**B**, **C**) were determined in KS tests. Crossbars are distributions' means. (**D**) RT-qPCR analysis of *LMNB1* and *LBR* gene expression in the same cells. *P*-values were determined in paired *t*-tests.

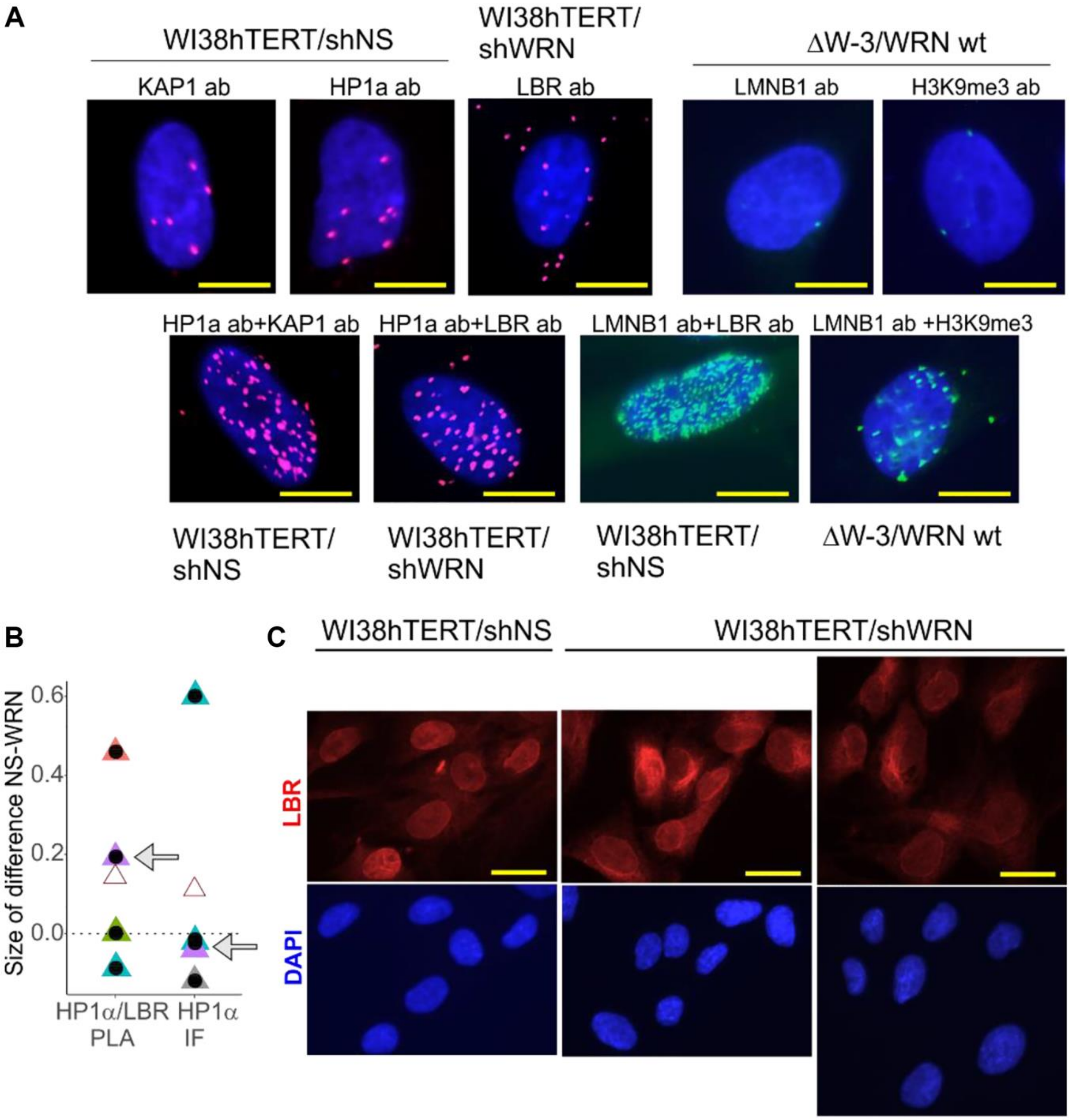

Supplementary Figure 6. (**A**) Examples of PLA results for the indicated single antibody controls and the corresponding pairwise antibody combinations. Red or green fluorescent mixes for PLA were used in these assays. Scale bar, 10 µm. (**B**) Sizes of differences between the datasets derived in HP1α/LBR PLA or HP1α IF assays for the control and WRN-depleted WI38hTERT cells were determined by calculating Cliff's delta statistic. Positive Cliff's delta values indicate overall lower values in WRN-deficient cells. Each independent experiment is marked by a different color and open triangles are means for all experiments. Grey arrows mark the values derived from the experiments shown in Figure 6A and 6B. (**C**) Examples of LBR subcellular distributions in WI38hTERT expressing the indicated shRNA. Scale bar, 20 µm.

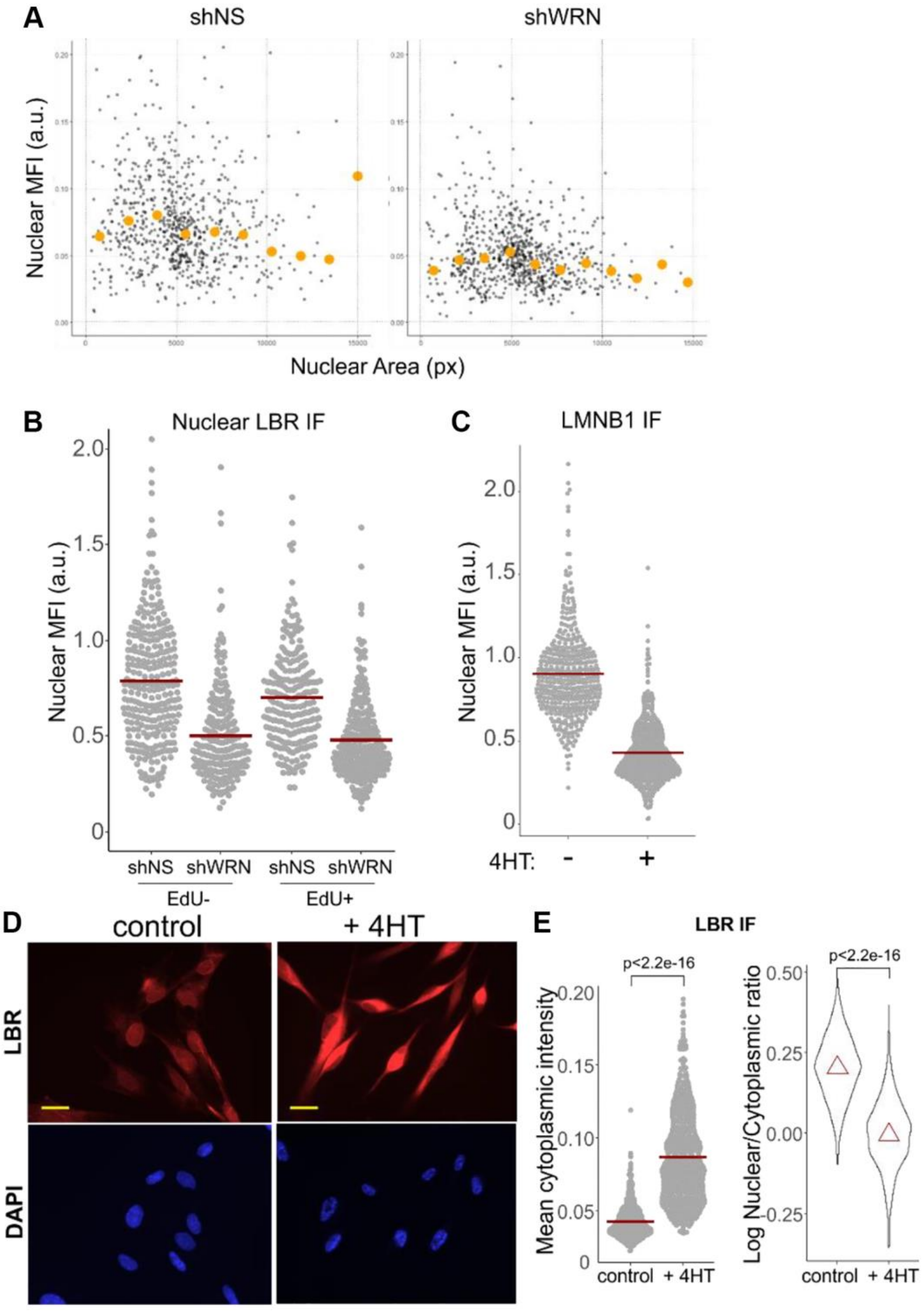

Supplementary Figure 7. (**A**) Nuclear LBR is reduced in the whole population of WRN-depleted cells including S phase cells. Nuclear LBR MFIs quantified in the control and WRN-depleted WI38hTERT were plotted as a function of nuclear area (in pixels). In addition, the distributions were binned by nuclear area, and mean LBR MFI was determined for each bin and plotted (orange circles). (**B**) The same cells as in (**A**) were labeled with 20 µM EdU for 30 min, fixed, and the incorporated EdU was clicked to Alexa-488 azide. Nuclear LBR MFIs were quantified and plotted separately for EdU+ and EdU- cells. (**C**) Reduction of nuclear Lamin B1 levels in WI38hTERT undergoing RAF-dependent OIS. (**D**) Changes in subcellular distribution and levels of LBR in the same cells. Scale bar, 20 µm. (**E**) Quantitation of data shown in (**D**), measuring distributions of cytoplasmic levels of LBR (per cell, left panel), and nuclear to cytoplasmic ratio of LBR MFIs (per cell, right panel) in OIS cells versus controls.

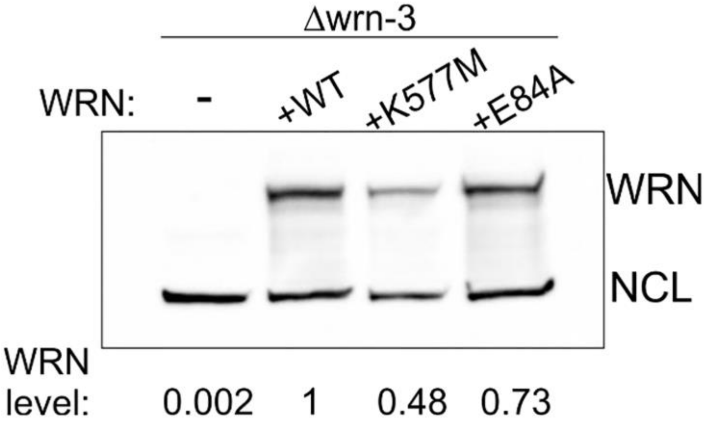

Supplementary Figure 8. Levels of WRN protein in whole cell extracts of Δwrn-3 GM639 cell line transfected with the indicated wild type and mutant WRN constructs. A Western blot was probed with the indicated antibodies. Values below the image are WRN levels normalized to the NCL loading control and shown relative to those in the wild type WRN-expressing cells.
